# Molecular and Evolutionary Bases of Within-Patient Genotypic and Phenotypic Diversity in *Escherichia coli* Extraintestinal Infections

**DOI:** 10.1371/journal.ppat.1001125

**Published:** 2010-09-30

**Authors:** Maxime Levert, Oana Zamfir, Olivier Clermont, Odile Bouvet, Sylvain Lespinats, Marie Claire Hipeaux, Catherine Branger, Bertrand Picard, Claude Saint-Ruf, Françoise Norel, Thierry Balliau, Michel Zivy, Hervé Le Nagard, Stéphane Cruvellier, Béatrice Chane-Woon-Ming, Susanna Nilsson, Ivana Gudelj, Katherine Phan, Thomas Ferenci, Olivier Tenaillon, Erick Denamur

**Affiliations:** 1 INSERM U722 and Université Paris 7 Denis Diderot, Faculté de Médecine, Site Xavier Bichat, Paris, France; 2 Assistance Publique - Hôpitaux de Paris, Hôpital Louis Mourier, Laboratoire de Microbiologie, Colombes, France; 3 INSERM U1001 and Université Paris 5 René Descartes, Faculté de Médecine, Paris, France; 4 Unité de Génétique Moléculaire and CNRS URA2172, Institut Pasteur, Paris, France; 5 CNRS UMR 0320/UMR8120 Génétique Végétale, Plate-Forme de Protéomique PAPPSO, Gif-sur-Yvette, France; 6 INSERM U738 and Université Paris 7 Denis Diderot, Faculté de Médecine, Site Xavier Bichat, Paris, France; 7 Laboratoire de Génomique Comparative, CNRS UMR8030, Institut de Génomique, CEA, Genoscope, Evry, France; 8 Department of Mathematics, Imperial College, London, United Kingdom; 9 School of Molecular Bioscience, University of Sydney, Sydney, New South Wales, Australia; Imperial College, United Kingdom

## Abstract

Although polymicrobial infections, caused by combinations of viruses, bacteria, fungi and parasites, are being recognised with increasing frequency, little is known about the occurrence of within-species diversity in bacterial infections and the molecular and evolutionary bases of this diversity. We used multiple approaches to study the genomic and phenotypic diversity among 226 *Escherichia coli* isolates from deep and closed visceral infections occurring in 19 patients. We observed genomic variability among isolates from the same site within 11 patients. This diversity was of two types, as patients were infected either by several distinct *E. coli* clones (4 patients) or by members of a single clone that exhibit micro-heterogeneity (11 patients); both types of diversity were present in 4 patients. A surprisingly wide continuum of antibiotic resistance, outer membrane permeability, growth rate, stress resistance, red dry and rough morphotype characteristics and virulence properties were present within the isolates of single clones in 8 of the 11 patients showing genomic micro-heterogeneity. Many of the observed phenotypic differences within clones affected the trade-off between self-preservation and nutritional competence (SPANC). We showed in 3 patients that this phenotypic variability was associated with distinct levels of RpoS in co-existing isolates. Genome mutational analysis and global proteomic comparisons in isolates from a patient revealed a star-like relationship of changes amongst clonally diverging isolates. A mathematical model demonstrated that multiple genotypes with distinct RpoS levels can co-exist as a result of the SPANC trade-off. In the cases involving infection by a single clone, we present several lines of evidence to suggest diversification during the infectious process rather than an infection by multiple isolates exhibiting a micro-heterogeneity. Our results suggest that bacteria are subject to trade-offs during an infectious process and that the observed diversity resembled results obtained in experimental evolution studies. Whatever the mechanisms leading to diversity, our results have strong medical implications in terms of the need for more extensive isolate testing before deciding on antibiotic therapies.

## Author Summary

We investigated whether an infection is a site of pathogen within-species diversity. Our results indicate that there is indeed extensive diversity during human extraintestinal infections by *Escherichia coli*. This diversity was of two types, not mutually exclusive, as we found that patients were infected either by several distinct *E. coli* clones or by members of a single clone that exhibit micro-heterogeneity. The high degree of phenotypic diversity, including antibiotic resistance, suggests that there is no uniform selection pressure leading to a single fitter clone during an infection. We discuss a possible mechanism and a mathematical model that explains these unexpected results. Our data suggest that the evolution of diversity in the course of an infection and in *in vitro* experimental evolution in the absence of host immune selective pressure may have many parallels. Whatever the mechanisms leading to diversity, our results have strong medical implications in terms of the need for more extensive isolate testing before deciding on antibiotic therapies.

## Introduction

Polymicrobial infections, caused by combinations of viruses, bacteria, fungi and parasites, are being recognised with increasing frequency [1]. Polymicrobial infections due to bacteria of the same species have been less studied, as they are clearly more difficult to identify. However, recent molecular epidemiological tools allow their systematic detection, as well as the study of the relatedness between strains. Two kinds of within-species diversity have been reported: polyclonal diversity with infection caused by phylogenetically divergent clones and monoclonal diversity with infection by members of a single clone that exhibit genetic micro-heterogeneity. Such within-species polymicrobial infection have been reported mainly in chronic and/or open infections (*Pseudomonas aeruginosa* infections of lung in cystic fibrosis patients [2], [3] and of burn wounds [4], *Helicobacter pylori* gastric infection [5], *Staphylococcus epidermidis* joint prosthesis infection [6] and endocarditis [7], lung tuberculosis [8]) but also in septicemia [9], [10].

Beside these clinical observations, *in vitro* experimental evolution has provided some clues on the diversification process in a single clonal population. It has been shown that stable polymorphisms can emerge due to ecological interactions [11], [12], [13], [14]. More detailed analysis of variation with larger numbers of sampled co-evolving bacteria showed that a very complex polymorphism with considerable phenotypic diversity could emerge in a matter of days [15], revealing a kind of adaptive radiation. In this case, selection under nutrient limitation altered the SPANC balance, or the trade-off between self-preservation and nutritional competence [16]. Polymorphisms affecting the SPANC balance often result in altered cellular levels of the sigma factor RpoS, which results in modification of several phenotypes, such as nutritional abilities, general stress resistance, starvation survival, cell morphology [17]. RpoS is a central regulator of stress resistance. Strains with higher RpoS level are more resistant to external stress but metabolised fewer substrates whereas strains with lower RpoS level have broader nutritional capabilities but lower resistance to external stress. Experimental evolution has also studied cooperation and competition among single species bacteria according to relatedness [18]. Overall, many different ecological sources of polymorphism have been identified in more than 60 years of bacterial experimental evolution. Yet, few studies have connected the molecular bases of such polymorphism to the one observed in the course of an infection. Currently, it is impossible to distinguish whether bacterial adaptation in the course of an infection is the action of the immune system [19] or the kind of process observed in *in vitro* experimental systems. Are the selective pressures identified as a source of diversification in *in vitro* experimental systems actually relevant during an infection?

*Escherichia coli* is a commensal of the intestinal tract of vertebrates, including humans [20], that can cause intestinal (diarrhoea) and extraintestinal [urinary tract infection (UTI), pneumonia, neonatal meningitis, septicaemia] infections [21]. Human extraintestinal infections have high incidence and associated morbidity, mortality (500,000 estimated deaths a year worldwide), and costs [22]. *E. coli* species can be considered as having mainly a clonal genetic structure [23], [24] with four main phylogenetic groups (A, B1, B2 and D) [25], [26]. Numerous epidemiological and animal model studies have documented the association of specific “virulence” determinants and/or phylogenetic groups with the different clinical syndromes [21], [27], [28]. It is classically admitted that *E. coli* extraintestinal infections are caused by identical isolates originating from single clones. From this assumption, the identification and antibiotic testing are mainly based on the characterisation of a single colony from the pathological specimen isolation. However, as for other bacterial species [2], [3], [4], [5], [6], [7], [8], [9], [10], this assumption is questionable.

In this context, we studied the level of genomic and phenotypic polymorphism of *E. coli* in human severe and closed extraintestinal infections. The aim of our work was (i) to get a better appreciation of the occurrence of within-species diversity in closed infections, (ii) to decipher the molecular and evolutionary bases of this diversity and (iii) to test, using a mathematical model, whether SPANC balancing can be a contributing factor to the observed pathogen diversification.

## Results

### Two kinds of intra-patient *E. coli* genetic diversity were observed: polyclonal and monoclonal with micro-heterogeneity

To have a global distribution of the genetic diversity of the 226 *E. coli* isolates from 19 patients (Table 1), we used a combination of phylogenetic grouping, multi locus sequence typing (MLST), Enterobacterial Repetitive Intergenic Consensus (ERIC)-PCR and pulsed field gel electrophoresis (PFGE) analyses to characterise the two kinds of diversity, *i.e.* polyclonal and monoclonal with micro-heterogeneity. Polyclonal infections were identified by distinct sequence type and/or phylogenetic group/subgroup and unrelated ERIC-PCR and PFGE patterns whereas monoclonal infection with micro-heterogeneity were characterised by identical sequence type and phylogenetic group/subgroup and distinct but related patterns of ERIC-PCR and PFGE [29].

**Table 1:** Main characteristics of the *E. coli* studied.

Patient ID^a^	Origin	Number of isolates	Phylogenetic group/subgroups^b^	Genetic micro-heterogeneity^c^	Virulence factor pattern variability^d^	Number of antibiotypes	Growth defect^e^
1	urine + blood	10+10	D_1_	−		1	
2	urine + blood	10+10	B2_3_	−		1	
3	hepatic abscess + blood	10+10	B2_3_	+		2	+
4	placenta + blood	20+10	B2_3_	−		1	
**5**	perirectal abscess	7	A_1_ f (5)A_1_ (2)	+−		11	+
6	hepatic abscess	8	A_1_	−		1	
7	ascitic fluid	8	D_1_	−		1	
**8**	bile	7	A_0_ (2)A_1_ (5)	++		12	
9	pleural fluid	10	B2_2_	−		1	
10	cerebrospinal fluid	5	A_1_	−		1	
11	cerebrospinal fluid	7	A_0_	+		2	+
12	urine	6	A_1_	+		2	
13	urine	7	B2_3_	+		2	+
14	pleural fluid	8	B1	+	*iucC*, *iutA*, *traT*	4	+
**15**	hepatic abscess	14	B1(2)B2_3_ (12)	−+		11	+
**16**	appendiceal abscess	9	A_1_ (1)B1 (7)B2_3_ (1)	−+−		131	
17	peritoneal fluid	9	A_1_	+	*iucC*, *iutA*, *traT*	2	+
18	urine	15	B2_3_	−		1	
19	urine + blood	5+11	D_1_	+	*iucC*, *iutA*, *traT*	2	+

a Bold numbers correspond to patients infected by distinct clones (polyclonal infections).

b Determined as in [63]. When several group/subgroups are present within a single patient, the number of isolates belonging to each group/subgroup is indicated in parentheses.

c Determined by PFGE and/or ERIC-PCR.

d Only variable virulence factors are indicated. All these variable genes are plasmid-located.

e A plus sign indicates that some isolates within a single patient exhibit impaired growth as compared to other isolates of the same patient.

f These two groups of A_1_ strains were differentiated by MLST.

**Figure 1:**
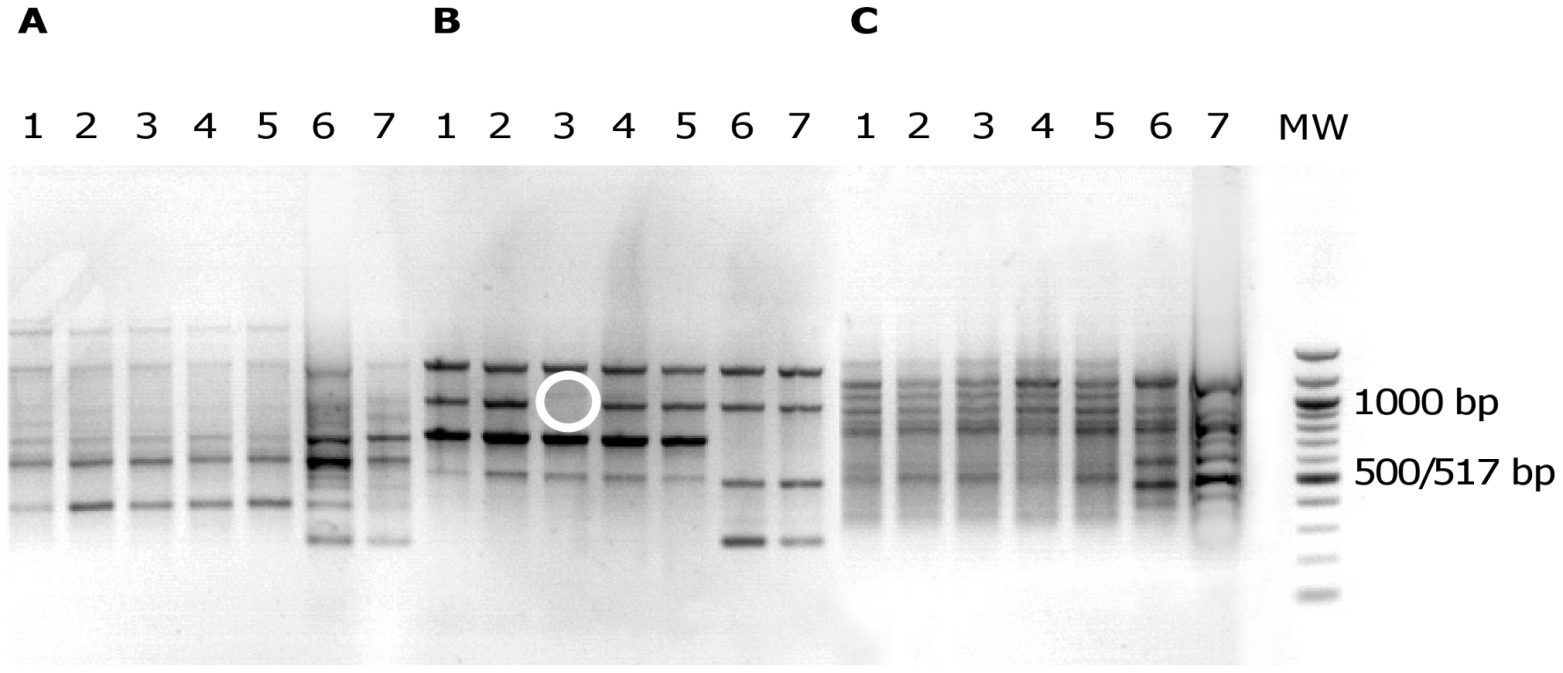
Electrophoretic patterns in agarose gel of PCR products from *E. coli* isolates. Primers ERIC1 (A), ERIC1R (B) and ERIC2 (C) were used for *E. coli* isolates of patient 5 (lanes 1 to 7). MW, DNA ladder (New England Biolabs, Saint Quentin en Yvelines, France). The banding patterns of isolates 1 to 5 are clearly different from the banding patterns of isolates 6 and 7, whatever the primer. This corresponds to two distinct *E. coli* clones. Of note, isolate 3 with primer ERIC1R (B) can be distinguished from other isolates of the same clone by one band (white circle). This corresponds to a genetic micro-heterogeneity.

**Figure 2:**
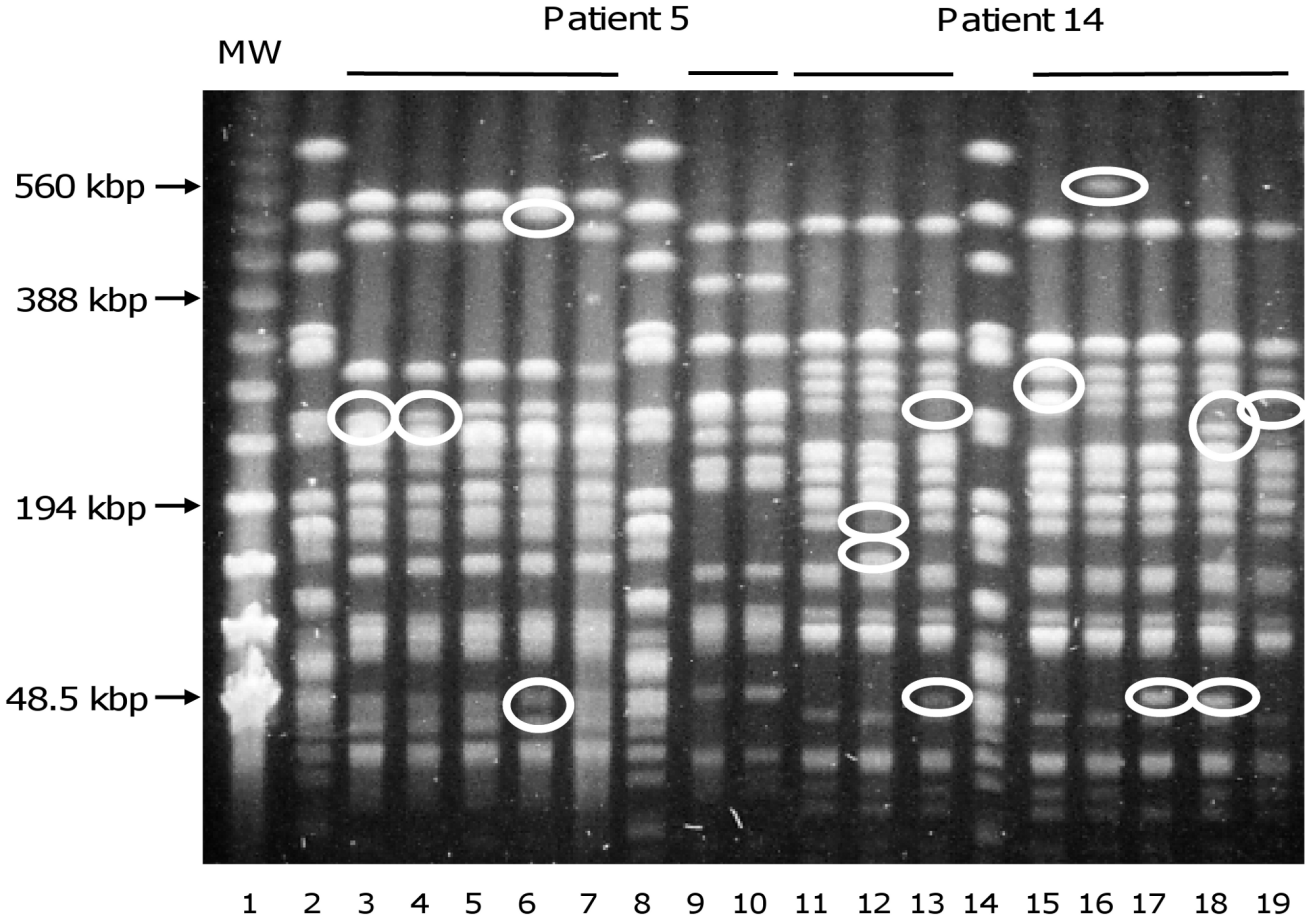
Pulsed-field gel electrophoresis resolving *Xba*I restriction fragments from *E. coli* isolates. Lines 3–7, 9, 10 and 11–13, 15–19 correspond to isolates of patients 5 and 14, respectively. Line 1, MW, DNA ladder (New England Biolabs, Saint Quentin en Yvelines, France), lines 2, 8 and 14, unrelated *E. coli* strain used as control. Differences between closely related strains are indicated by circles. Patient 5 was infected by two different *E. coli* clones (lines 3–7 and 9,10). Banding patterns of lines 3–7 are closely related, with differences affecting only one or two bands, but completely different from banding patterns of lines 9 and 10, which are identical. These data are in agreement with the ERIC-PCR data (Fig. 1). The isolates of patient 14 (lines 11–13, 15–19) are closely related with seven bands being variable among all isolates.

**Figure 3:**
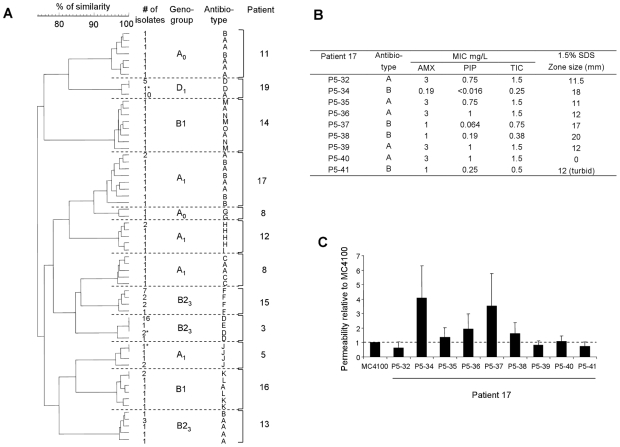
Antibioresistance phenotypes of *E. coli* isolates. (A) Dendrogram constructed from the pulsed-field gel electrophoresis patterns of 104 isolates of *E. coli* showing genetic micro-heterogeneity in 12 clones infecting 11 patients with the antibiotypes. The antibiotypes are indicated with the corresponding isolates as in Table S2. Ward's algorithm was used to cluster the isolates from a similarity matrix created by using the band-based Dice similarity coefficient. The stars indicate the isolates differentiated by ERIC-PCR. Patient 8 is infected by one A_1_ clone and one A_0_ clone. Abbreviation of the antibiotypes is as follow: A, susceptible to all antibiotics tested; B, susceptible to all antibiotics tested and hyper- susceptible to β-lactams; C, Penicillinase production; D, Penicillinase production and resistant to tetracycline (Te), minocycline (Mn), sulfonamides (Sul), streptomycin (St), kanamycin (Ka); E, resistant to Te, Mn, Ka; F, resistant to Te, Mn, Sul, St; G, Penicillinase production and resistant to Te, Mn, Ka; H, Penicillinase production and resistant to Te, Sul, trimethoprim (TMP), St, Ka, nalidixic acid (Na), chloramphenicol (Ch); I, resistant to Te, Sul, St, Na, Ch; J, Penicillinase production and resistant to Te, Mn, Sul, TMP, St; K, Penicillinase production and resistant to Te, Mn, Ch; L, Penicillinase production and resistant to Te, Mn, Sul, St, Ch; M, resistant to Sul, St; N, resistant to Te; O, resistant to Te, Sul, St. Various level of susceptibility to penicillin antibiotics were observed among isolates of patient 17. (B) Minimum inhibitory concentration (MIC) of three β-lactams [amoxicillin (AMX), Piperacillin (PIP), Ticarcillin (TIC)] and susceptibility to SDS of the nine isolates from patient 17. The antibiotype is as in (A). Note the heterogeneous patterns of MICs and SDS susceptibility of the hyper-susceptible isolates to β-lactams, indicating the implication of various mechanisms of increased outer membrane permeability. (C) Outer membrane permeability rates for the nine isolates from patient 17, compared to the rate for *E. coli* K-12 strain MC4100. The permeability rate for MC4100 was 3.96, in units of optical density change at 492 nm/min/10^10^ bacteria.

In 8 cases (42%) corresponding to 3 UTI, 1 meningitis, 1 pleural infection, 1 ascites, 1 placenta infection and 1 hepatic abscess (patients 1, 2, 4, 6, 7, 9, 10 and 18), no genetic diversity was observed (Table 1). In patients 5, 8, 15 and 16 (21%) (Table 1), corresponding to intra-abdominal infections, polyclonal infections were observed. In patient 16, three different clones corresponding to 3 distinct phylogenetic groups were observed among the isolates. In patients 8 and 15, the isolates belong to 2 different phylogenetic group/subgroups (Table 1). In patient 5 where the two isolates belong to the A_1_ subgroup (Table 1), MLST analysis clearly showed two distinct sequence types corresponding to a large divergence time. Among these infections, isolates exhibited different ERIC-PCR patterns with each of the three primers (see Fig. 1 for an example). Moreover, these 4 patients with polyclonal infections have at least one of the clone exhibiting micro-heterogeneity (Table 1), characterised by closely related but different patterns observed by ERIC-PCR with only one or two primers and by PFGE (see Fig. 1 and 2 for an example). In the remaining 7 patients (3, 11, 12, 13, 14, 17 and 19), monoclonal infections with micro-heterogeneity were detected by ERIC-PCR and PFGE (Table 1). In these cases of micro-heterogeneity, the isolates belong to the same phylogenetic group/subgroup and sequence type using 7 essential genes as well as the sequence of the *gnd* gene, one of the most variable genes in *E. coli* [30]. Altogether, presence of at least a clone with micro-heterogeneity was observed in 11 patients (58%). A dendrogram constructed from the PFGE patterns of *E. coli* isolates from these patients is shown in Fig. 3A. Within a patient, the level of similarity between isolates is above 94% (Fig. 3A). In patients 3 and 19 from which blood and an additional sample were available, the micro-heterogeneity was observed in both samples. Lastly, all the 19 patients were infected by unrelated clones (Table 1 and Fig. 3A).

The presence of 20 virulence factors (VFs) known to be associated to extra intestinal virulence [31] and representative of the main classes of VFs (protectin, adhesin, toxin, iron uptake system) were studied. A wide range of VF patterns was observed from the absence of detected gene in A group strains to the presence of 12 studied genes in the B2 group strains, in agreement with the well known link between virulence and phylogeny [27] (data not shown). Among the 4 patients (5, 8, 15 and 16) infected by several distinct clones, the VF patterns of each clone within a patient were always different. In 3 patients infected by a clone exhibiting a micro-heterogeneity (14, 17 and 19), the *iucC* and *iutA* genes, associated to *traT* gene, were either present or absent, indicating the variable presence of a plasmid bearing the aerobactin operon (Tables 1 and S1).

In summary, a high level of genetic diversity was frequently observed in the isolates originating from a single patient. Two kinds of genetic diversity were observed: (i) polyclonal infection and (ii) monoclonal infection with micro-heterogeneity. These two kinds of genetic diversity can be combined in a single patient.

### Isolates from a single clone with genetic micro-heterogeneity exhibit variable antibiotic susceptibility and growth patterns

**Figure 4:**
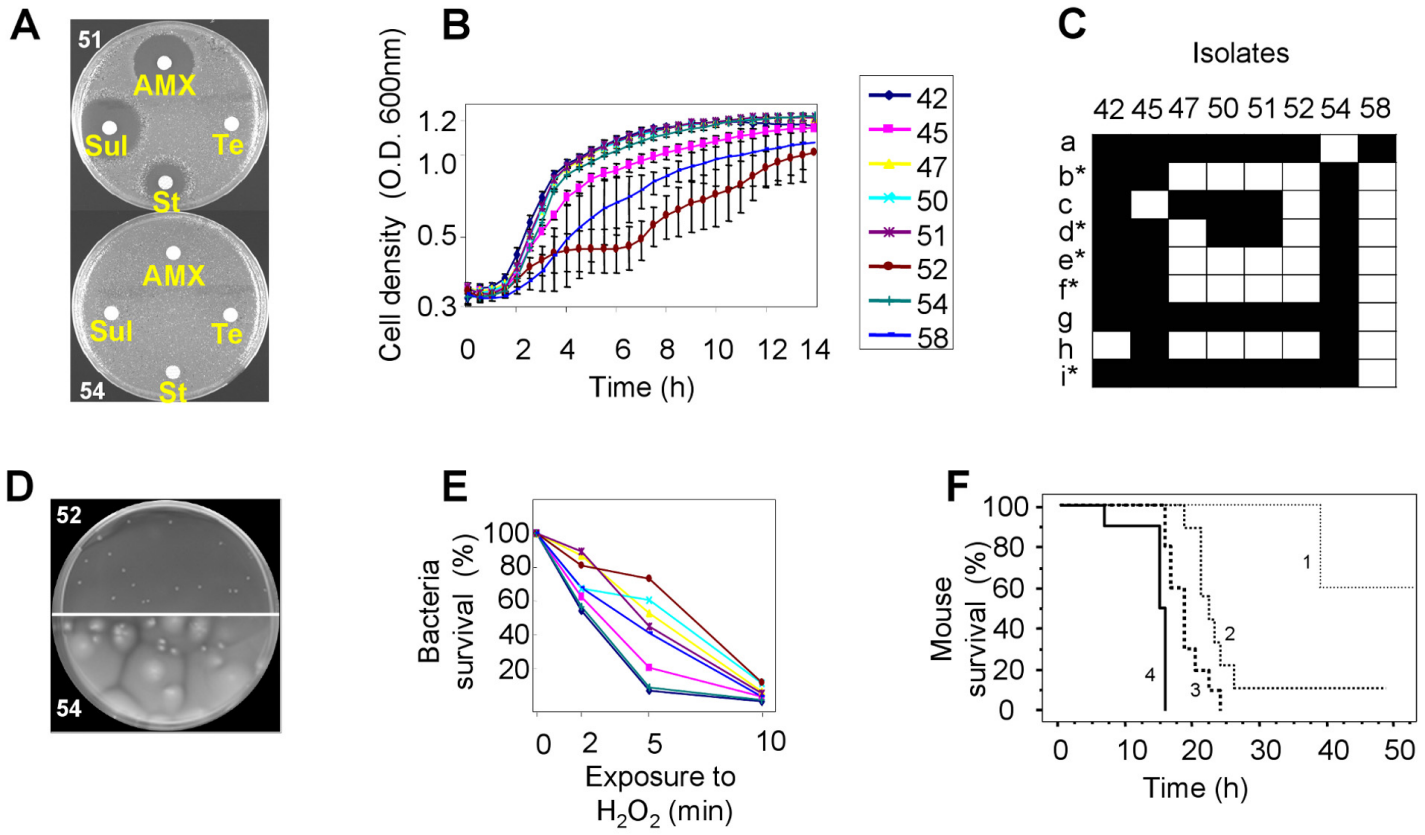
Illustration of the phenotypic diversity observed in the *E. coli* isolates of patient 3 infected by a unique clone [isolates from blood (ID 42–50) and liver abscess (ID 51–58)]. (A) Variability in the antibiotypes with a plasmid loss in isolate 51 responsible for the susceptible phenotype. AMX: amoxicillin, Sul: sulfonamide, St: streptomycin, Te: tetracycline. (B) Growth curves in Luria Bertani broth at 37°C of 8 isolates, showing impaired growth of isolates 45, 52 and 58. (C) Carbon source utilisation using Biolog GN2 plates that test 95 substrates. Only substrates differentially used by the isolates are indicated. The substrates are as follows. a: Dextrin, b: Bromosuccinic Acid, c: Glucuronamide, d: L-Alanine, e: L-Asparagine, f: L-Aspartic Acid, g: Glycyl-L-Aspartic Acid, h: L-Serine, i: D,L-α-Glycerol Phosphate. Substrates with a star correspond to substrates whose metabolism is stimulated by an *rpoS* disruption [39]. Black and white squares indicate the use or the absence of use of the substrate, respectively. (D) Non motile (52) and motile (54) isolates in 0.35% agar plates incubated for 48 hours at 37°C in humid atmosphere. (E) Sensitivity to H_2_O_2_ measured by the survival in % at different times. The greatest differences were observed at 5 minutes. The colour code for the isolates is as in (B). (F) Kaplan-Meier curves estimating the survival function of mice subcutaneously inoculated by 2×10^8^ colony forming units of the different isolates. Only one curve of each statistically significant category is presented. Isolates range from the more to the less virulent as follow: isolates 54 and 50 (group 4), isolates 42, 47 and 51 (group 3), isolates 52 and 58 (group 2) and isolate 45 (group 1).

We then wanted to know if the genetic micro-heterogeneity observed within isolates of a single clone was associated with phenotypic variation. To this purpose, we studied in all the isolates two phenotypes having critical impact on strain fitness in an infection, *i.e.* antibiotic sensitivity and growth. Among the 226 *E.coli* isolates, 19 distinct antibiotypes were distinguished. We found 9 clones in 9 patients (3, 8, 11, 12, 13, 14, 16, 17 and 19) exhibiting heterogeneity in the antibiotic resistance pattern, all of which having a genetic micro heterogeneity (Tables 1 and S2, Fig. 3A and 4A). The antibiotics concerned were the β-lactams, tetracycline, streptomycin and kanamycin, the sulfonamides, trimethoprim and chloramphenicol. Within these single clones, 2 to 4 distinct antibiotypes were evidenced, the most sensitive phenotype being always observed in the minority of the isolates (Table S2). Several causes of variation in antibiotic susceptibility were likely. Firstly, loss of genetically mobile resistance genes was confirmed by a perfect correlation between the β-lactam resistance phenotype and the positive TEM (β-lactamase) PCR results in the isolates from 5 patients (3, 8, 12, 16 and 19). This mechanism is also likely involved in the loss of resistance to tetracycline, streptomycin, kanamycin, sulfonamides, trimethoprim and chloramphenicol, as the genes encoding for these resistances in clinical isolates are located on mobile elements such as transposons and integrons [32]. Patients 14 and 16 are particularly illustrative of this phenomenon as multiple combinations of antibiotic resistance losses were observed within a single patient (Table S2). Secondly, hyper-susceptibility to β-lactams was observed in 3 patients (11, 13 and 17). We have further studied the isolates of patient 17. Susceptibility to antibiotics (amoxicillin, piperacillin and ticarcillin) and detergents (SDS) was not constant and outer membrane permeability assays indicated heterogeneity in permeation rates due to variations in porin-mediated diffusion (Fig. 3B). Thirdly, the discrepancy between outer membrane permeability (Fig. 3C) and antibiotic/SDS susceptibility (Fig. 3B) points to yet other differences in envelope structure or efflux between isolates [32], suggesting an unexpectedly extensive range of surface variations inside a patient.

Even more strikingly, heritable variation in growth characteristics was found amongst clonal isolates from 8 patients, all infected by at least one clone with genetic micro-heterogeneity (Tables 1 and 2). Impaired growth was characterised by longer lag time and/or generation time and/or reduced biomass in stationary phase (Table 2). Within a patient, impaired growth was found in 6 to 22% of isolates (mean 13%), the remaining isolates having identical unimpaired growth kinetics (Fig. 4B and Tables 1 and 2).

**Table 2:** Phenotypic characteristics of *E. coli* isolates showing an impaired growth.

Isolate ID^a^	Growth^b^			Biolog GN2^c^	Motility^c^	H_2_O_2_ sensitivity^c^	Acid sensitivity^e^
	Lag time (h)	Generation time (h)	Biomass in stationary phase (10^8^ bacteria.ml^−1^)				
3-42	1.5 (0)	1.7 (0.03)	14.4 (0.2)	39	+	+	intermediate
3-45	1.5 (0)	2.4 (0.09)	13.9 (0.3)	39	+	+	intermediate
3-47	1.5 (0)	1.7 (0.06)	14.8 (0.3)	35	+	−	low
3-50	1.5 (0)	1.6 (0.05)	14.9 (0.4)	36	+	−	low
3-51	1.5 (0)	1.6 (0.07)	15 (0.4)	36	+	−	low
3-52	1.5 (0.2)	4.7 (0.36)	11.4 (1.4)	34	−	−	low
3-54	1.5 (0)	1.7 (0.09)	15.1 (0.5)	38	+	+	intermediate
3-58	2.0 (0.1)	4.3 (0.41)	12.2 (2.2)	32	−	−	low
5-4228	2.3 (0)	1.8 (0.04)	12.6 (0.1)	48	+	+	intermediate
5-4225	2.3 (0)	1.8 (0.04)	10.4 (0.4)	44	−	−	intermediate
11-4252	1.5 (0)	1.5 (0.01)	14.1 (0.1)	ND^d^	+	−	low
11-4250	1.8 (0)	2.0 (0.03)	13.3 (0.2)	ND	+	+	high
13-4432	2.2 (0)	1.8 (0.1)	13.7 (0)	40	+	−	intermediate
13-4433	2.6 (0)	2.3 (0.01)	12.9 (0.2)	35	−	+	high
14-4231	1.9 (0)	1.6 (0.03)	13.9 (0.1)	ND	−	+	low
14-4245	2.6 (0)	2.0 (0.09)	11.5 (0.2)	ND	+	−	intermediate
15-P5-30	1.9 (0.1)	1.4 (0.07)	14.0 (0.2)	43	+	+	intermediate
15-P5-24	2.2 (0)	1.9 (0.11)	13.3 (0.2)	40	−	−	low
17-P5-40	1.9 (0)	1.7 (0.05)	13.7 (0)	37	ND	+	intermediate
17-P5-38	2.4 (0)	1.8 (0.04)	13.5 (0.1)	28	−	−	intermediate
17-P5-41	2.7 (0)	3.3 (0.56)	13.9 (0.1)	34	+	−	intermediate
19-486	2.3 (0.2)	1.3 (0.07)	14.4 (0.2)	45	+	+	low
19-474	2.4 (0)	1.6 (0.04)	13.2 (0.2)	43	−	−	low

**^a^**First number corresponds to the ID patient. The first isolate is the normal growth control.

**^b^**The experiments were repeated 3 times. The data are presented as the mean +/− standard deviation in brackets.

**^c^**In all cases, comparisons are made between isolates of the same patient. For the Biolog GN2, the total number of used carbon sources is indicated. See Table S4 for the complete list of carbon sources. For motility and H_2_O_2_ sensitivity, only two categories were considered: + corresponds to increased motility and H_2_O_2_ sensitivity as compared to −.

**^d^**ND, not determined because of the absence of growth.

e See material and methods for the categorization.

### Commensal clonal populations are phenotypically less diverse than pathogenic ones

A crucial question is whether micro-heterogeneity is due to multiple infections of micro-evolved genotypes of the same clone from a common route and/or source of infection or to evolution during the infectious process. We thus compared within-clone phenotypic diversity in the 19 populations isolated from infections and in 15 clonal populations isolated from the faeces of 15 healthy subjects matched for geographic origin and sex with the infected patients (10 colonies studied per sample). We found in healthy subjects one change of antibiotic profile within a subject, affecting the resistance to sulfonamides in 7 isolates, and one change in growth ability within another subject, affecting one isolate from 10. This is a significantly different pattern as compared to the infected patients (Wilcoxon two sided test, *p*  =  0.015), consistent with increased genetic differentiation during infection.

### Mutators are present within monoclonal isolates of a unique patient

A way to increase the genetic and phenotypic diversity is to increase the mutation rate. We thus measured the frequencies of mutations conferring resistance to rifampin in the isolates of 9 patients where a strain with genetic micro-heterogeneity was identified. In patient 14 the isolates were all resistant to rifampin. The median value of mutagenesis for the 91 isolates from the remaining 8 patients was 3.33×10^−9^, a value not different from the previously reported *E. coli* collections [33]. Considering a threshold of 10-fold the median value for defining mutators, 3 isolates were mutators: one in patient 3, one in patient 12 and one among the A_1_ isolates in patient 17 (Table S3). Among these three isolates, one from patient 17 displayed a >50-fold increase in mutagenesis and was considered as a strong mutator. Such strong mutators have been shown to be essentially mismatch repair deficient in the wild [34].

Isolates exhibiting a micro-heterogeneity did not have a higher mutation rate than commonly found in the species *E. coli*. Hence, increased mutation rate should not be the primary cause of the observed phenotypic diversity, yet the presence of some mutator isolates at quite high frequency compared to their expected production by mutation, suggests that some strong adaptation might be under way in those populations [35].

### The level of RpoS is variable within isolates of a single clone

In environments as in the host where stress and nutritional competition are both important, selection may well affect SPANC-related phenotypes [15], [36], [37].

**Figure 5:**
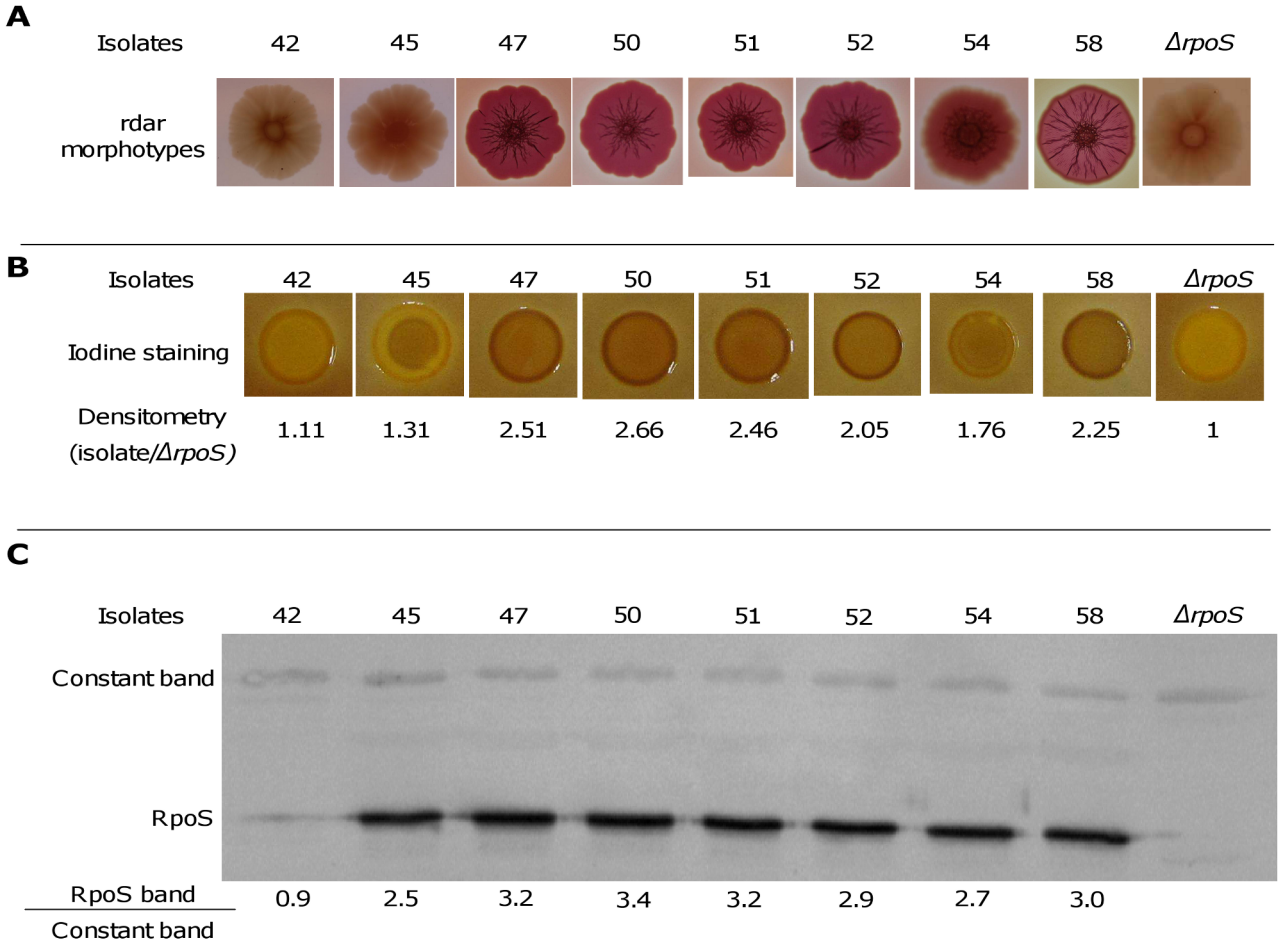
Level of RpoS in 8 representative *E. coli* isolates of patient 3. The level of RpoS was assessed by rdar morphotypes (A), staining glycogen with iodine solution (B) and RpoS immunoblot (C). RpoS amount is expressed as the ratio of the RpoS band to a constant cross-reactive band. The negative control (*E. coli* MG1655 Δ*rpoS* strain) is on the right part of the figure. Experiments in B and C were repeated 2 times, given values are the mean of the two experiments.

**Figure 6:**
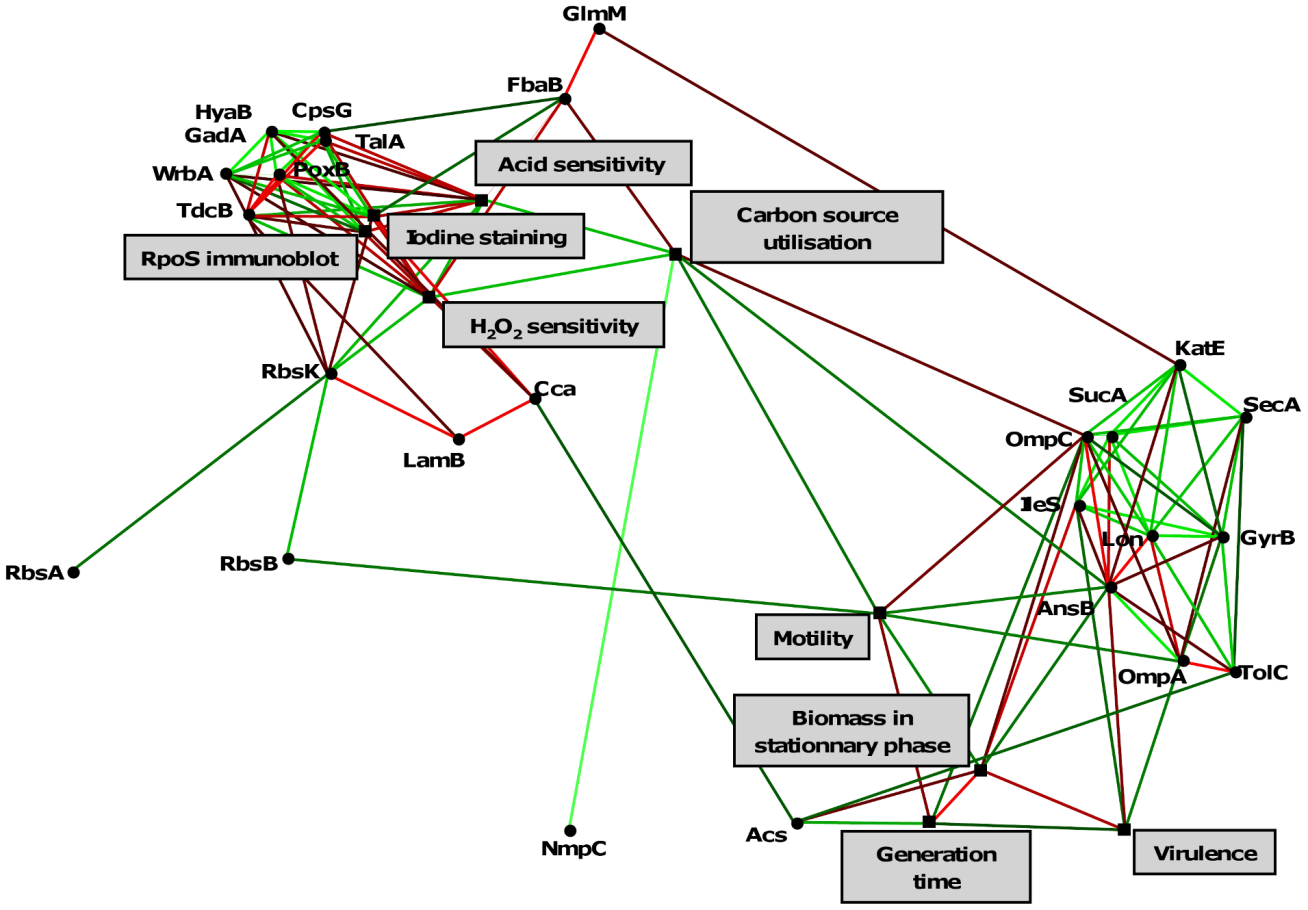
Statistically significant links between phenotypes and the 27 differentially expressed proteins of the 8 representative *E. coli* isolates of patient 3. The links depicted by a line, in green when positive (phenotypes and/or amounts of protein increase together) or in red when negative (a phenotype or an amount of protein increases when the other increases). Lighter is the line, more significant is the link. Only one protein (putative outer membrane protein) is not linked. Note that the links presented here are coherent with those of the phenotypes from the data set corresponding to 23 isolates originating from 8 patients (Fig. S1).

We studied the variability of the SPANC-related phenotypes in the 8 patients showing differences in growth kinetics. Using an assay that tests the metabolic capacity of a strain to use a wide array of carbon sources (Phenotype MicroArrays) [38], we found that isolates from a single clone within a single patient have distinct patterns of substrate use (Fig. 4C and Tables 2 and S4). Interestingly, 9 of these substrates differentially used within isolates from a single patient (Table S4) are among the 13 substrates whose metabolism is stimulated by an *rpoS* disruption [39]. Motility and sensitivity to H_2_O_2_ and acid were also highly variable among isolates from a single patient (Figs 4D and E and Table 2). Thus, heterogeneity in motility pattern was evidenced in 7 of the 8 patient isolates, with the majority of isolates within a patient being motile and the minority being non motile whereas polymorphism in the H_2_O_2_ and acid sensitivity was observed in all and 5 patients, respectively. Furthermore, a significant relation was observed between growth, capacity to use the substrates, sensitivity to H_2_O_2_ and acid and motility. Strains showing impaired growth use fewer substrates, are more resistant to H_2_O_2_ and acid but less motile (Fig. S1), in accordance with the proposed SPANC trade-off [39]. We also studied the development of the red dry and rough (rdar) morphotype, which is depending of RpoS [40]. This morphotype is a multicellular behavior characterized by expression of the adhesive extracellular matrix components curli fimbriae and cellulose [41]. The morphotypes within isolates of single patients were highly variable (see Fig. 5A for an example). To confirm the role of RpoS in the observed polymorphism, we studied in more detail the 8 isolates of patient 3, the 7 isolates of the patient 13 and the 9 isolates of patient 17. The level of RpoS in the cell was assessed qualitatively by staining for presence of glycogen (RpoS regulates positively *glgA*, the glycogen synthase gene) and quantitatively by RpoS immunoblotting using specific antibodies. Both assays were consistent and showed isolate-specific endogenous levels of RpoS (Figs 5 and S2 and data not shown). Although the variations were not drastic except for few isolates, the fact that they were highly correlated between both techniques as well as to the nutritional and stress phenotypes and the rdar morphotypes, and the differentially expressed proteins for the patient 3 (see below and Fig. 6), is an argument for their physiologic relevance. Hence a major determinant of the polymorphism observed appear to be linked to the SPANC balance as observed in some experimental evolution settings.

### Twenty-seven proteins are differentially expressed within the isolates of a single clone in relation to the SPANC-related phenotypes

To decipher the molecular bases of the RpoS phenotype, we first sequenced the *rpoS* promoter and gene but surprisingly did not detect any mutations amongst the 8 representative isolates of patient 3. We then performed differential proteomics on these 8 isolates by bidimensional electrophoresis (2-DE) and mass spectrometry. Twenty-seven proteins were significantly differentially expressed between the isolates, with a 1.8 to 24 fold range (Fig. S3 and Table 3). Among them, 16 were involved in central metabolism, 9 were membrane proteins and 2 were stress proteins. Furthermore, 14 out of the 27 differentially expressed proteins were known to be RpoS regulated (Table 3). Within the 8 isolates, the levels of the 27 proteins, except one putative outer membrane protein, were significantly related, as well as to the level of RpoS, the sensitivity to H_2_O_2_ and acid, the capacity to use substrates, the motility and the growth, with one group of proteins closely linked to the level of RpoS and the other with growth (Fig. 6).

**Table 3:** List of the 27 proteins differentially expressed between the 8 representative *E. coli* isolates of patient 3.

Protein	Gene^a^	Fold change^b^	ANOVA (*p*)
Central metabolism			
Ribokinase	*rbsK*	8.26	9.25E-08
Fructose biphosphate aldolase class I	fbaB*	4.91	1,46 E-3
Pyruvate dehydrogenase	poxB*	24.2	8,56E-04
Acetyl-coA synthetase	acs*	2.90	7,58E-04
Hydrogenase 1 large subunit	hyaB*	20	3.48E-04
NAD(P)H:quinone oxidoreductase	wrbA*	8.24	5.78E-04
Transaldolase A	talA*	6.15	1,83E-04
2-oxoglutarate dehydrogenase subunit	sucA*	19.7	2.97E-05
Phosphomannomutase	*cpsG*	4.40	6.20E-04
Phosphoglucosamine mutase	*glmM*	1.76	4.85E-05
Periplasmic L-asparaginase II	*ansB*	5.83	9.31E-06
Threonine/Serine deaminase	*tdcB*	3.19	2.29E-05
DNA-binding ATP-dependent protease	*lon*	8.11	7.30E-10
DNA gyrase, subunit B	*gyrB*	9	2.68E-06
tRNA nucleotidyl transferase	*cca*	2.13	8.27E-04
Isoleucyl-tRNA synthetase	*ileS*	4.25	4.21E-05
Membrane			
D-ribose transporter subunit	*rbsA*	20.0	4.11E-06
D-ribose transporter subunit	rbsB*	14.3	6.63E-07
Preprotein translocase subunit, ATPase	*secA*	6.79	9.03E-04
Transport channel	*tolC*	2.13	3.17E-04
Outer membrane protein A	ompA*	9.03	8.73E-05
Outer membrane protein C	ompC*	4.65	1.13E-05
Maltose outer membrane porin	lamB*	3.4	8.57E-04
Outer membrane porin; DLP12 prophage	nmpC*	1.87	7.90E-04
Putative outer membrane protein	ND^c^	6.78	8.10E-05
Stress			
Glutamate decarboxylase A, PLP-dependent	gadA*	4.37	4.98E-05
Catalase HPII	katE*	3.50	8.77E-04

a Genes indicated by a star were shown to be regulated by RpoS [83], [84], [85], [86], [87].

b Calculated between minimum and maximum normalized spot volumes of the 8 isolates.

c ND, not determined.

### Few mutations are associated with the phenotypic changes observed within the isolates of a single clone

**Figure 7:**
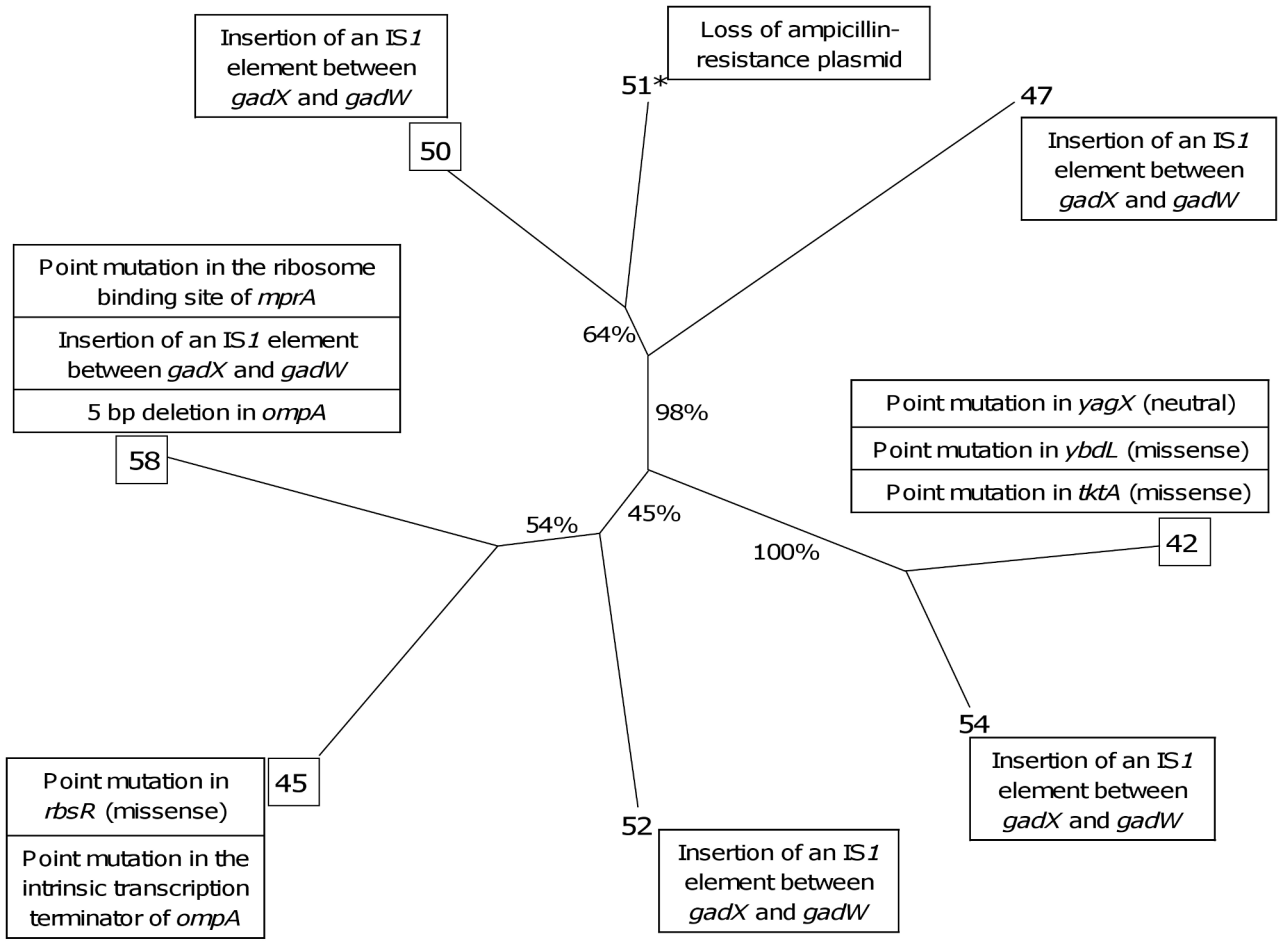
Neighbour joining distance tree based on the phenotypic and proteomic data indicating the relationships between the 8 representative *E. coli* isolates of patient 3 with the identified mutations. Bootstrap values in percentages are calculated from 10,000 replicates. The boxed isolates correspond to the isolates fully sequenced. The isolate with a star (51) is a mutator isolate. The identified mutations (Table 4) as well as the plasmid loss are indicated in boxes at each isolate.

To identify the mutations responsible for these phenotypic changes, we performed whole-genome sequencing of 4 (42, 45, 50 and 58) of these 8 isolates of the patient 3 and searched by PCR for the identified mutations in the 4 (47, 51, 52 and 54) remaining isolates. Six point mutations, one small deletion and one IS insertion were identified, generating a star like phylogeny with each of the four sequenced isolates having its own combination of mutations (Fig. 7 and Table 4). The point mutations in 42, 45 and 58 isolates were absent from the remaining isolates in Fig. 7. The IS insertion was only identified in two of four sequenced isolates, but was shown by PCR to be in 47, 52 and 54 isolates as well. The genes affected were encoding for metabolic functions, including the acid resistance system dependent upon glutamate, and for membrane proteins. Of note, *ompA* was the target of 2 different molecular defects in isolates 45 and 58 (Table 4), which is a very strong sign of selection. A mutation in the repressor of the *rbs* operon involved in the D-ribose catabolic function, was observed. This operon was also shown to be the target of inactivating mutations selected during experimental evolution in glucose minimal medium [42]. Most of the mutations were corroborated by the proteomic changes in the corresponding isolate (Table 4).

**Table 4:** Mutations identified by whole genome sequencing on 4 *E. coli* isolates of patient 3.

Isolate	Localisation of the mutation	Mutation^a^	Function	Protein variation^b^
42	*yagX* *ybdL* *tktA*	G 384,100 A (Phe 118 Phe)T 676,307 C (Met 240 Thr)T 3,376,675 C (Ile 21 Val)	Aromatic compound dioxygenaseMethionine aminotransferaseTransketolase 1, thiamin-binding	
45	*rbsR*Intrinsic transcription terminator of *ompA*	A 4,440,121 C (Thr 288 Pro)C 1,062,754 A	DNA-binding transcriptional repressor of ribose metabolismOuter membrane protein	RbsA, B, K +OmpA −
50	Between *gadX* and *gadW*	Insertion of an IS*1* element at 4,103,497	Transcription regulators of *gadA*	GadA +
58	Ribosome binding site of *mprA*Between *gadX* and *gadW* *ompA*	T 3,091,307 CInsertion of an IS*1* element at 4,103,4975 bp deletion between 1,063,473–1,063,479	DNA-binding transcriptional repressor of multidrug efflux pump EmrAB-TolCTranscription regulators of *gadA*Outer membrane protein	TolC +GadA +OmpA −

a The numbers between nucleotides correspond to the localisation of the mutation in the CFT073 genome according to MaGe [79] whereas the number between the amino acids corresponds to the amino acid numbering of the protein, when relevant.

b The variation is given for the protein itself or for proteins directly regulated by the encoded protein. Plus and minus signs indicate increased or decreased level of protein, respectively. Data are from the proteomic analysis (Table 3).

### Multiple genotypes with distinct levels of RpoS can co-exist as a result of the trade-off

Can SPANC balancing provide an explanation for *E. coli* diversity observed in the clonal isolates of a single patient described above? To test this hypothesis we consider a mathematical model that is built on a series of simple assumptions regarding bacterial metabolism and the environment in which bacteria reside, but that allows us examine in isolation the role of SPANC balancing in the creation and maintenance of diversity.

In our model we assume that *E. coli* isolates differ in their RpoS expression, *x*, normalized to *0*≤*x*≤*1* so that an *rpoS*- mutant has *x = 0* and the *rpoS* ^+^ with a maximal level of resistance has *x = 1*. Evolutionary changes in *x* are constrained by the SPANC balance trade-off in the following way: an increase in *x* leads to a decrease in the maximal resource uptake rate denoted by a decreasing function *f(x)*; an increase in *x* also leads to an increase in the stress protection denoted by an increasing function *c(x)*. Next we consider an *E. coli* population with *n* competing isolates each with a different value of the RpoS expression *x*, and define 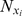 to be the density of an isolate with phenotype *x_i_* where *i = 1…n* and *0* = *x_1_*≤*x_2_*≤*…*≤*x_n_ = 1*. We assume that mutations altering *x* occur at a rate ε taking into account both mutations in known regulators of RpoS levels as well as mutations that pleiotropically affect the levels of RpoS. A schematic representation of the model can be found in Fig. S4.

**Figure 8:**
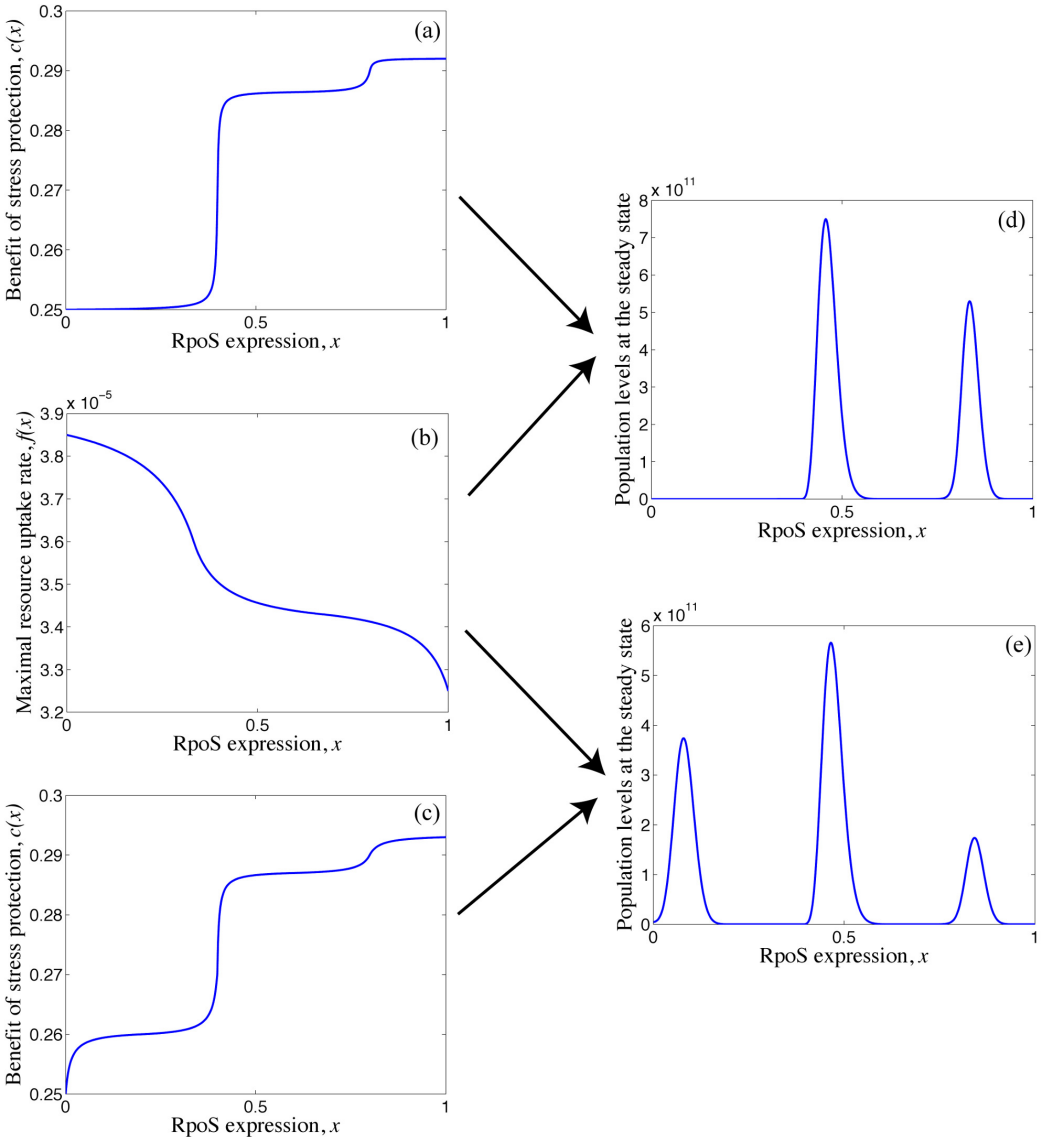
Model of the distinct levels of RpoS. (d) Long-term population structure supporting two genotypes with distinct levels of RpoS, requires the benefit of stress protection and maximal rate of resource uptake to have the form illustrated in (a) and (b) respectively. (e) Long-term population structure supporting three genotypes with distinct levels of RpoS, requires the benefit of stress protection and maximal rate of resource uptake to have the form illustrated in (c) and (b) respectively. Here ε = 0.325×10^−8^, *S_0_*  =  1.1×10^9^ picomoles of sugar, *D* = 0.0017 per minute, *k* = 4×10^6^ picomoles of sugar and *r* = 237 cells per picomole of sugar. The maximal uptake rate *f(x)* is measured in picomoles of sugar per cell per minute.

Solving numerically the system of equations (1) we find that starting with an isogenic *rpoS*+ population, the equilibrium population can support two or more partial *rpoS-* mutants depending on the shape of the SPANC balance trade-off namely on the shape of *f(x)* and c*(x)* (Fig. 8). For example, if mutations, that further decrease the levels of RpoS in a type that already has low levels of RpoS, do not lead to a marked decrease in stress protection (Fig. 8a), the long-term population structure supports two genotypes with distinct RpoS levels (Fig. 8d). On the other hand if mutations, that further decrease the levels of RpoS in a type that already has low levels of RpoS, lead to a clear decrease in stress protection (Fig. 8c), the long-term population structure supports three genotypes with distinct levels of RpoS (Fig. 8e).

Note that in our model the coexistence of multiple genotypes requires the assumption that changing RpoS levels from 1 to 0 induces three distinct levels of stress protection (Fig. 8a and c). While to our knowledge the shapes of the SPANC balance trade-off have not been determined in detail these forms are not unrealistic because stress responses to most environmental stresses involve not just RpoS, but also additional stress-specific responses (*e.g.* the heat shock or low pH responses) whose expression is directly or indirectly affected by the RpoS level. For example the acid stress resistance is influenced by numerous components including AR2 system whose expression can be initiated with either of two sigma factors RpoS and RpoD [43]. Similarly, DsrA, a small RNA, influences multiple acid resistance genes of *E. coli* as well as RpoS levels [44]. Therefore changing levels of DsrA, AR2 repressor H-NS and RpoS could give rise to non-linear relationships in the SPANC balance trade-offs as illustrated in Fig 8.

This simple model demonstrates that the SPANC balance is sufficient to create diversity in RpoS expressions within an *E. coli* population, and does not require any specific effect of the immune system.

### The intrinsic virulence of isolates from a single clone is highly variable

Even if models predict that the mutants with variable levels of RpoS may emerge in the absence of immune selective pressure, we wanted to test the impact of RpoS phenotype on virulence. We therefore investigated the intrinsic extraintestinal virulence of each of the 8 representative isolates of patient 3, in a mouse model of septicemia [27]. Statistically significant differences were obtained between isolates, defining 4 groups (1 to 4), with the most virulent killing 100% of the mice in less than 15 hours (group 4) and the least virulent killing only 40% of the mice (group 1), the 2 other groups being intermediate (Fig. 4F). These groups are significantly correlated to growth properties; better growth gives greater virulence (Fig. 6). In confirmation, competition experiments in mice inoculated with a 1/1 ratio of isolate 58 (having an impaired growth) and reduced mouse killing (group 2) and isolate 42 (having a normal growth) and relatively high mouse killing (group 3) (Fig. 4F), showed a survival curve identical to the one observed in mice infected with isolate 42. Bacterial counts in the spleens of the killed mice showed a culture consisting > 99% of the 42 isolate. Identical results between these two strains were obtained after 24 h of *in vitro* competition in Luria Bertani broth (data not shown).

Hence the RpoS-associated impaired growth is not beneficial in the systemic phase of infection mimicked by the mouse model of septicemia, and the polymorphism observed may be driven mostly by the SPANC balance.

## Discussion

### Infections of a single patient by several distinct isolates is common in extraintestinal *E. coli* infections and result from different phenomena

To our knowledge, this work is the first one to investigate thoroughly, using molecular tools, numerous isolates from single patients in a large series of deep and severe extraintestinal *E. coli* infections. *E. coli* responsible for extraintestinal infections can be considered as opportunistic pathogens with the commensal reservoir being the intestinal tract [20], [21]. We observed an unexpectedly high level of within patient bacterial polymorphism as 11 of the 19 (58%) patients were infected by genotypically and/or phenotypically diverse bacteria. This diversity, as previously reported in other species [2], [3], [4], [5], [6], [7], [8], [9], [10], is of two types. Patients are infected either by several distinct *E. coli* clones or by a single clone with micro-heterogeneity. Both types of diversity can be observed in some patients. Patients with distinct *E. coli* clones were clearly infected from the intestinal commensal niche by different clones. Of note, these multiple infections are physically closely related to the commensal reservoir (Table 1). For the patients infected by a single clone that presents some micro-heterogeneity, two scenarios, not mutually exclusive, can be envisaged: the observed diversity could have pre-existed to the infection process in which a subset of the digestive tract diversity would have been sampled or the diversity could have emerged in the course of the infection that was initiated by a single isolate or few identical isolates. Several arguments favour the second hypothesis. First, in the cases with micro-heterogeneity, the infected organs were more diverse (kidney in 3 cases, digestive apparatus in 2 cases, pleural fluid and cerebrospinal fluid in one case each, associated in 2 cases to bacteremias) than in the patients infected by distinct clones, suggesting a less evident route for digestive tract bacteria to the infection zone. Second, we have studied the level of phenotypic polymorphism in *E. coli* isolates from stools of healthy subjects harbouring unique clones and found a significantly lower level of polymorphism in antibiotic resistance and growth patterns than in infections. Third, the tree reconstructed from the phenotypic and proteomic data (Fig. 7) of the 8 representative isolates of the patient 3 appears as a star like phylogeny and indicates a rapid diversification from a unique ancestor. Fourth, such scenario of diversification during the infectious process has been experimentally confirmed in a rat model of *S. epidermidis* foreign body-associated infection [7]. Similarly, genomic changes were observed in *H. pylori* during gastric experimental infection in rhesus monkey [45]. Lastly, it has been demonstrated in immunocompetent rodent models that bacteremias resulting from experimental colonisations are the products of very few bacteria [46], [47], [48], or even a single one [49].

### The molecular bases of the within-clone phenotypic diversity

Some of the observed phenotypic diversity (antibiotic resistance, iron capture system) was easily explained by the loss of plasmid borne determinants. It has been shown that in stressful conditions, the induction of the SOS system mobilises transposons [50], integrons [51], as well as virulence genes [52]. The remaining phenotypic diversity was mainly RpoS related but we did not identified mutation in the *rpoS* gene or in the more than 20 diverse regulators that have been shown to influence RpoS levels [17]. Instead, we observed few mutations in metabolic and membrane related genes (Table 4). The convergence in *ompA*, the mutation in the *rbs* operon that has been shown to be under selection in experimental evolution system [42] and the presence of mutators (Table S3) are strong arguments for the fact that these mutations have been selected. Of note, *ompA* is among the 23 genes that show evidence of positive selection in *E. coli* [53]. The genomic mutations we identified suggest that SPANC balance can be affected by an even larger set of pleiotropic mutations, many of them yet unknown. For example, the overproduction of the EmrAB-TolC multi-drug efflux transport system in the *mprA* mutant, and the nutrient starvation in the *ompA* mutants could explain the altered growth and the RpoS response in isolates 45 and 58 (Table 4). In these cases, the RpoS phenotype would be secondary to the phenotype generated by the mutations. A limitation of our approach is that, (i) we could have missed IS based chromosomal reorganisations and gene amplification(s) and (ii) unlike in experimental evolution experiments, we do not have the ancestor clone that could allow us to perform reconstruction experiments with the mutated alleles and competitions [54], confirming the direct implications of the mutations in the observed phenotypes.

### The evolutionary forces that drive the single clone diversity

A wide continuum of growth rate, stress resistance, outer membrane permeability, rdar morphotype and virulence properties were observed within isolates of single clones, directly linked to the SPANC trade-off and associated with distinct levels of the sigma factor RpoS in isolates (Figs 3, 4, 5, 6 and S1). *rpoS* mutations have been reported in *E. coli* laboratory and natural isolates as they are probably selected for in nutrient-limited environments [16]. Indeed, an important variation in the level of RpoS was observed in an *E. coli* clonal population grown in constant environment in a chemostat. This population radiated in more than five phenotypic clusters, two including *rpoS* mutant isolates [15]. In some other experimental evolution systems, duplications of *rpoS* have been reported [37], suggesting that higher or lower level could be achieved depending on the exact setting of the experiment. Our data in infected patients hence parallel these observations made in experimental evolution systems in the absence of immune selective pressure, and were explained by the simple mathematical model. This fully supports the idea that the SPANC balance is sufficient to promote a strong phenotypic diversification at intermediate stress levels (Fig. 8). Of course there are numerous other mechanisms [55] that could generate the diversity patterns observed in this study, such as environmental heterogeneities or immune system driven negative-frequency dependence. But the model provided here is a test of an alternative coexistence mechanism. The model assumptions have been deliberately kept simple in order to illustrate a minimal set of conditions under which the SPANC balance trade-off could lead to coexistence of *E. coli* genotypes with differing levels of RpoS. In particular the SPANC balance trade-off is defined through a single evolving phenotype, namely the RpoS expression and we conclude that complex but nonetheless realistic shapes of the trade-off are required for the diversity to be maintained. However, increasing the number of traits through which the trade-off exists will reduce the complexity of the shapes required to observe diversity [56].

The lack of mutations in *rpoS* itself that we observed suggests RpoS loss is detrimental under host infection conditions. Indeed, the role of RpoS *in vivo* is not clear as *rpoS* mutants are selected within the gut in commensal *E. coli* [57] but are avirulent in a mouse model of septicemia with *Salmonella* [58]. To test further if RpoS polymorphism is a virulence-linked effect, we tested the various isolates of patient 3 in a mouse model of infection. Yet we could not identify a virulence-linked benefit of high level of RpoS, as lower virulence was associated to lower growth rate (Fig. 4F and 6).

Another source of variation supported by theoretical and experimental approach is the one concerning iron uptake. Iron is essential for the growth of bacteria and they produce and release iron-chelating small molecules known as siderophores to scavenge iron from their hosts. Siderophore production is considered as an altruistic trait that is costly for the individual but provides a group benefit because other individuals can take up the siderophore-iron complex [18]. *P. aeruginosa* mutants that do not produce siderophore (cheaters) have been seen to evolve both *in vitro* [59] and in the lungs of cystic fibrosis patients [60]. Similarly, we observed within clone variability due to the coexistence in a same sample of isolates with different siderophore encoding gene content due to plasmid loss (Table S1). A frequent observed polymorphism in the monoclonal infection cases is also the presence or absence of penicillinase production (Fig. 3A and Table S2), due to a *bla* plasmid borne gene. As in the case of siderophore production, penicillinase production could be seen as an altruistic trait in the presence of antibiotics. The effect could also be reinforced by the fact that plasmids may carry other secreted (altruistic) proteins [61].

One emerging question related to the presence of some strong diversity in phenotypes as important as growth, resistance to stress or antibiotic resistance, is what are the consequences in term of the infection outcome. In other words, how such a polymorphism affects the virulence of the bacterial population. The emergence of cheaters lacking siderophore or eventually antibiotic resistance genes (and the other genes carried on the plasmid) clearly illustrates that selection may not always favour the most virulent genotypes, and that within-population selection may on the opposite select for genotypes that reduce the infectious power of the population as a whole [62]. Along the same line, our mathematical modelling suggests that the SPANC trade-off allows the emergence of some variable bacteria independently of the host immune system, and our animal model of infection revealed that the most stress resistant ones are not the more virulent. Yet, the existence of some strong variability along the SPANC balance may sometimes be beneficial as many different strategies are present within the population and some may be more efficient than other in the different stages of the infection.

### Concluding remarks

Our results suggest that experimental evolution and the evolution of diversity in the course of an infection may have many parallels. Whether or not this diversity promotes increased virulence remains an open question, that will require further work to link the observed mutations to the various phenotypes and to some potential virulence linked effects.

Whatever the mechanisms leading to diversity (infections by several distinct isolates or diversification during the infectious process), our results have strong medical implications in term of antibiotic therapies. Indeed, although an individual can harbour several independent commensal clones [63], the classical medical assumption about extraintestinal *E. coli* infections is that they are strictly monoclonal. This is typified by the antimicrobial susceptibility testing performed on few (2 to 5) colonies obtained from the pathological sample to establish antibiotic treatment [64], [65]. This may fail to detect the presence of other, more resistant isolates, and lead to therapeutic failure [66]. Our work showing an impressive level of antibiotic resistance phenotype variability within the isolates of a single patient argues for the utilisation of a mix of numerous isolates to perform the antibiogram, or, alternatively for the realisation of simplified antibiograms with essential molecules on numerous isolates in addition to the classical antibiogram on a single isolate.

## Materials and Methods

### Bacterial strains

A total of 226 *E. coli* isolates responsible for severe infections in 19 epidemiologically unrelated adult patients from 3 university hospitals in France [Brest, Paris suburbs (Colombes and Clichy)], were studied (Table 1). To avoid contamination, only deep and closed visceral infections were selected (pyelonephritis, meningitis, pleurisy, cholecystitis, intra-abdominal deep abscess, ascitic fluid, peritoneal fluid and placental infections and bacteraemia). For 5 patients (1, 2, 3, 4 and 19), two samples were analysed (blood plus another site of infection), whereas only one sample was studied for the remaining patients. Except for urinary tract infection, samples were obtained during surgery or through sterile punctures. In all these infections, *E. coli* alone was recovered. For each sample, 5 to 20 colonies isolated on blood agar or CLED (cystine lactose electrolyte deficient) plates were randomly selected and stored with glycerol at −80°C before use. In addition, 150 commensal isolates belonging to 15 subjects (10 isolates per subject) matched for geographic origin (Brest, Paris suburbs) and sex with the patients [63] were studied as controls. Each subject exhibits identical isolates based on the phylogenetic group/subgroup and the virulence gene content, but the isolates of the different subjects belong to various phylogenetic group/sub groups. Isolates were stored with glycerol at −80°C before use.

### Human ethics statement

All the sampling procedures of the infected patients were performed in the course of the clinical diagnosis. No additional procedure was performed in the patients for the present study. The study was approved by the institutional ethics committee (Comité de Protection des Personnes, Hôpital Saint-Louis, Paris; #2004-06). For the healthy subjects, the study was approved by the ethics committee of INSERM (IRB0000388, FWA00005831), with the opinion #01-014. In both studies, the participants were informed of their role in the study and written informed consent was provided by study participants.

### PCR phylotyping and virulence factor pattern

All pathogenic isolates were assigned to the 7 *E. coli* phylogenetic group and subgroups using triplex PCR based on the presence or absence of three DNA fragments (*chuA*, *yjaA* and TSPE4.C2) [63]. The presence of 20 extraintestinal virulence factors (VFs) (*neuC*, *sfa*, *iroN*, *iucC*, *iutA*, *iha*, *papC*, *papG*, *hlyC*, *cnf1*, *hra*, *sat*, *ire*, *usp*, *ompT*, *ibeA*, *malX*, *fyuA*, *irp2* and *traT*) was tested by PCR on all isolates, as previously described [31].

### Enterobacterial Repetitive Intergenic Consensus (ERIC)-PCR

ERIC-PCR was performed on all pathogenic isolates, as previously described [67] using separately three different primers to increase the discriminatory power of the technique: ERIC1 (5′-GTGAATCCCCAGGAGCTTACAT-3′), ERIC1R (5′-ATGTAAGCTCCTGGGGATTCAC-3′) and ERIC2 (5′-AAGTAAGTGACTGGGGTGAGCG-3′).

### Pulsed Field Gel Electrophoresis (PFGE) and analysis of strain relatedness

One hundred and ten pathogenic isolates from 11 patients (3, 5, 8, 11, 12, 13, 14, 15, 16, 17 and 19) in whom strain heterogeneity was observed in the antibiotypes and/or phylogenetic group/subgroups and/or virulence gene patterns and/or ERIC patterns, were further studied by PFGE. As previously described [68], PFGE was performed after digestion of chromosomal DNA with *Xba*I, which gave a convenient number of fragments. These fragments were separated in 0.5 X TBE buffer, pH 8.0, at 14°C and 6 V/cm with pulse times of 5–50 s for 21 h. DNA patterns obtained were analysed with Gel Compar software (Applied Maths, Kortrijk, Belgium). A similarity matrix was created by using the band-based Dice similarity coefficient, and Ward's algorithm was used to cluster the strains.

### Multi Locus Sequence Typing (MLST)

MLST analysis based on the concatenated sequences of 6 essential genes (*trpA*, *trpB*, *pabB*, *putP*, *icd* and *polB*) [26] was performed in some cases to determine the relatedness between the isolates. In addition, the sequence of *gnd*, one of the most variable gene in *E. coli* due to a high level of recombination [30], was occasionally performed to further discriminate isolates.

### Antibiotic susceptibility

Antimicrobial susceptibilities were determined on all the pathogenic and commensal isolates by the method of disc diffusion, according to the guidelines of European Committee for Antimicrobial Susceptibility Testing (www.eucast.org). The tested resistance markers were as follow: amoxicillin (AMX), amoxicillin-clavulanate, ticarcillin (TIC), piperacillin (PIP), piperacillin-tazobactam, imipenem, cefalotin (Cf), cefamandole, cefuroxime, cefoxitin, cefotaxime, ceftazidime, cefepime, aztreonam, mecillinam, streptomycin (St), kanamycin (Ka), gentamicin, tobramycin, netilmicin, amikacin, tetracycline (Te), minocycline (Mn), nalidixic acid (Na), pefloxacin, ciprofloxacin, chloramphenicol (Ch), fosfomycin, sulfonamides (Sul), trimethoprim (TMP). MICs were determined by the E-test method (AB Biodisk, Solna, Sweden) as recommended by the manufacturer. The production of penicillinase (Pase) was deduced from the resistance profile (resistance to amoxicillin, ticarcillin, piperacillin and intermediate susceptibility to amoxicillin-clavulanate, piperacillin-tazobactam and cefalotin) and confirmed by the detection of *bla*_TEM_ by specific PCR amplification as previously described [69].

### Outer membrane permeability

The isolates of patient 17 were electroporated with the plasmid pBR322 to introduce high levels of β-lactamase into all strains. Outer membrane permeability was measured by comparing β-lactamase activity in whole cells (with intact outer membrane) to that in disrupted cells with no barrier, using the permeable colorimetric substrate nitrocefin [70]. Bacterial strains were grown to exponential phase at 37°C in Luria-Bertani (LB) broth without salt containing either 100 µg/ml ampicillin or 15 µg/ml tetracycline. The optical density of the culture was measured and two 100 µl samples were taken. One aliquot (whole cells) was washed once, resuspended in the same volume of 0.1× M9 and the second aliquot was centrifuged and resuspended in 30% sucrose, 33 mM Tris. 5 µl 2 mg/ml lysozyme and 5 µl 0.2M EDTA and the mixture placed on ice for 15 min. Lysed bacteria were centrifuged for 5 min at 4°C, the pellet discarded and 3 µl 1M MgCl_2_ added to the supernatant. The broken cells and whole cells were assayed in 96 well micro titre plates with each well containing 160 µl 0.1×M9, 20 µl 50 µg/ml nitrocefin and 20 µl of the sample. The 96 well plate reader was configured for kinetics at 492nm for 20 min with readings taken every 2 min. The activity of the broken cells was very similar and quadruple assays were used to generate the values plotted in Fig. 3C. The activity is presented as the rate of optical density change/min/cell in the assay.

### Bacterial growth

Cells grown overnight in LB broth were washed and transferred into a fresh LB medium at a dilution of 1∶250. The growth rates were then measured for all the pathogenic and commensal isolates at 37°C by monitoring the optical density at 600 nm (Tecan microplate reader) in LB medium. Experiments were repeated 3 times.

### Carbon source utilisation

Biolog GN2 (Biolog, Inc., Hayward, CA) microplates were used to detect carbon utilization of 95 substrates in isolates of patients where a micro-heterogeneity was observed. Utilization of various C sources is coupled to reduction of a tetrazolium dye and production of purple colour [38]. Each strain was grown in LB medium, washed and resuspended to an optical density at 600 nm of 0.01 in mineral medium. Mineral medium was prepared as described elsewhere [38]. Plates were incubated at 37°C and colour changes were measured by optical density measurement (Tecan microplate reader) at a wavelength of 600 nm. The cut off point between negative results and positive results was an optical density of 0.2. Experiments were repeated 2 times. For the statistical analysis, the data considered were, for each isolate, the number of used substrates.

### Hydrogen peroxide sensitivity

Bacterial isolates from patients showing differences in the growth kinetic were grown overnight in LB, washed, and resuspended in 0.9% NaCl to a concentration of about 1.10^8^ cells ml-1. H_2_O_2_ was added to a final concentration of 50 mM. Aliquots of bacteria were removed at timed intervals, and numbers of viable cells were determined on LB plates. For the statistical analysis, only two categories were considered (resistant or sensitive). To delimit in categories the 8 isolates of patient 3, area between curves was used as a distance in order to compare curves. A k-means approach (two seeds, *i.e.* two clusters) on such distance matrix allows an automatic separation of clusters. As a control for the choice of the number of clusters, we represented these curves (according to the described distances) as points in a two dimensional Euclidean space thanks to an effective non-linear multidimensional algorithm named Data Driven High Dimensional Scaling (DD-HDS) [71]. Two clusters (categories) can be considered as fair.

### Acid sensitivity

To assess the acid sensitivity phenotype of the isolates from patients showing differences in the growth kinetic, we used a protocol previously described [72] with some minor modifications. An inoculum containing about 10^8^ cells from an overnight LB culture was introduced into LB acidified with HCl (35%) to pH 2.5. After two hours of exposure to pH 2.5, surviving cells were counted and the percentage of survivor was calculated. Strains which exhibited greater than 10% survival were designed acid resistant (low sensitivity), strains with 0.1% to 10% were designed intermediate in acid sensitivity, and those with less than 0.1% survival were considered acid sensitive (high sensitivity).

### Motility

Cells of isolates from patients showing differences in the growth kinetic were grown overnight in LB, washed, and resuspended in minimum media (PO_4_ acetate) to a concentration of 1.10^2^ cells ml^−1^. About 10 cells were grown in standard Petri dishes containing 20 ml minimum (PO_4_ acetate) media with 10mM glucose, solidified with 0.35% agar. Swim plates were incubated 48 hours at 37°C in humid atmosphere. Experiments were repeated 2 times. For the statistical analysis, only two categories were considered (motile or non motile).

### Rdar morphotype

Cells of isolates from patients showing differences in the growth kinetic were grown on LB agar plates without NaCl (LB0) at 28°C. We determined colony morphology and colour using LB0 agar supplemented with Congo red (40µg.ml-1) and Coomassie brilliant blue (20 µg.ml-1), as described previously [41].

### Iodine staining

Level of RpoS was obtained in 8 selected isolates of patient 3 by staining colonies on LB agar plates. Plates were incubated for 24 hours at 37°C and then left at 4°C for 20 hours before being flooded with Lugol (I_2_ concentration  =  10g.L^−1^). Iodine staining is dependent on glycogen, whose synthesis is affected by RpoS [73]. Dark-brown colonies correspond to a high level of RpoS. A control corresponding to *E. coli* MG1655 Δ*rpoS* strain was used. Experiments were repeated 2 times. For the statistical analysis, a scale from 1 to 5 depending on the darkness was used.

### RpoS immunoblot

Cells were harvested in stationary phase after 18 hours of culture in LB at 37°C, and proteins were extracted in SDS 1% at 100°C for 10 min. Protein concentration was measured by the method of Bradford (DC protein assay, Bio-Rad). SDS-PAGE was carried out in 10% polyacrylamide minigels (Mini Protean II; Bio-Rad). 10 µg of total proteins was loaded into each lane. The proteins were transferred to Amersham Hybond-P membrane (GE Healthcare) with a Mini TransBlot cell (Bio-Rad) in transfer buffer (25 mM Tris [pH 8.2], 192 mM glycine, 20% [vol/vol] ethanol). Western blot analysis was performed by serially incubating the membrane with polyclonal rabbit antibodies against the σS protein [74] overnight (1∶150000), and Amersham ECL anti-rabbit IgG (GE Healthcare), for one hour (1/20000). The RpoS protein was visualized using the Amersham ECL Plus Western Blotting Detection System (GE Healthcare). Quantifications were performed using a PhosphorImager (Molecular Dynamics). Data correspond to the ratio of the RpoS signal to a constant cross-reactive band. A control corresponding to *E. coli* MG1655 Δ*rpoS* strain was used. Experiments were repeated 2 times, values are the mean of the two experiments.

### *rpoS* gene and promoter sequencing

DNA was prepared from 8 selected isolates of patient 3 using the Wizard Genomic DNA purification Kit (Promega). Two PCR products (product 1: primer forward ACAAGGGGAAATCCGTAAACC; primer reverse AGGATTTCGCGCAAACG, length: 1059 bp, and product 2: primer forward CCGTACTATTCGTTTCGGCCGA; primer reverse GAGACTGGCCTTTCTGACAG, length: 536 bp) were used to obtain the sequence of the *rpoS* gene and its promoter. Sequencing of PCR products was performed on both strands by classical Sanger technology.

### Mouse extraintestinal virulence assay

A mouse model of systemic infection was used to assess the intrinsic virulence of 8 selected isolates of patient 3. For each isolate, 10 outbred female Swiss OF1 mice (3–4 weeks old, 14–18 g) received a 200 µL subcutaneous abdominal injection of 10^9^ CFU/mL of stationary-phase bacteria. Mortality was monitored for 7 days [27]. Kaplan-Meier curves were drawn from the data. Isolates were classified in 4 significantly different virulence groups, from group 1 for the less virulent to group 4 for the more virulent group (*p* of log rank test < 0.05). This classification was used for the statistical analysis. Competition experiments were performed by inoculating 10 mice by a 1/1 ratio of strains 42 and 58. Cells were numbered in the spleens of animals after grinding and sprawl on LB agar plates. Distinction between isolates 42 and 58 was done using the iodine staining assay that stains glycogen (RpoS regulates positively *glgA*, the glycogen synthase gene and colonies from isolate 58 were darker than colonies from isolate 42).

### Animal ethics statement

Animal experiments were performed in compliance with the recommendations of the French Ministry of Agriculture and approved by the French Veterinary Services (accreditation A 75-18-05).

### Mutation frequency

Mutation frequencies of the isolates from 8 patients (91 isolates) showing a micro-heterogeneity were estimated by monitoring their capacity to generate mutation conferring resistance to rifampin in at least three independent cultures for each isolate, as described previously [33].

### Bidimensional electrophoresis (2-DE) and mass spectrometry analysis

For each of the 8 representative isolates of patient 3, three independent protein extractions followed by 2-DE analysis were realised. Cells were harvested exactly at the same time at the beginning of stationary phase in LB and, after centrifugation washed three times in physiological serum. Proteins were extracted from cells by TCA-acetone precipitation according to [75], solubilized in R2D2 buffer [76] and quantified using the PlusOne 2-D Quant kit (Amersham Biosciences, Arlington Heights, IL). Isoelectrofocusing was carried out using 24-cm long, pH 4–7 Immobiline DryStrips (Amersham Biosciences) rehydrated in R2D2 solubilization buffer to which 300µg of protein extract was added. Full focusing was achieved after application of 84,000 V/hr at 20°C in a Protean IEF Cell (Bio Rad, Hercules, CA). Strips were equilibrated according to [77]. Second dimension electrophoresis was performed at 14°C (16 hr, 30 mA/gel) on a 24×24-cm gel (11% acrylamide, 2.9% of PDA crosslinker) in a Protean Plus Dodeca cell (Bio-Rad). Gels were stained by colloidal Coomassie blue and scanned using the PowerLook III scanner (Umax) and LabScan software (Amersham Biosciences). 2-DE gel image analysis was performed using Progenesis SameSpots (Nonlinear Dynamics). After optical density calibration, spot volumes were normalized according to total spot volume in each gel. Relative quantification of the detected spots was made in percentage of total spot volume (integrating optical density and spot area) for each gel, which allowed normalization of the values. Spots showing a significant variation between isolates (ANOVA test: p-value < 0.001) were excised with an EXQuest Spot Cutter (BioRad) and digested using a standard trypsin protocol as described by [78]. After digestion the supernatants were harvested and frozen at −20°C. Mass spectrometry identification of proteins was performed using a MALDI-TOF mass spectrometer Voyager DE super DTR system (Applied Biosystems, Foster City, CA, USA) equipped with a nitrogen laser emitting at 337 nm. Spectra were obtained in the mass range between 1000 and 2500 Da and were calibrated using internal calibration with autolytic trypsin fragments. Proteins were identified using the protein sequence database search program MS-FIT (http://prospector.ucsf.edu/prospector/4.0.7/html/msfit.htm) and the database MaGe (Genoscope, Evry France) [79]. Database search results were only accepted if the score reported by MS-FIT was higher than 1e4.

### Whole genome sequencing

Four representative of the 8 isolates of patient 3 were fully sequenced. Genomic DNA was prepared using the Wizard genomic DNA purification kit (Promega) and sequenced with Solexa (Illumina) technology (Genoscope, Evry, France). The fastq files were then analysed though the Sanger pipeline SSAH2 [80], using CFT073 genome [79] as a reference genome. Preliminary analysis showed that it was the closest *E. coli* sequenced genome. We first confirmed this observation as the isolates are less than 0.1% diverged from CFT073 and share more than 95% of their genome with CFT073. For each isolate, using the mapping of reads on the reference genome, we could identify positions at which the sequence differed from this reference sequence. At all these sites, the number of reads supporting each of the four bases was recorded in all the isolates. For each site, an exact Fisher test was then performed among all pairs of isolates to detect any difference in the distribution of the counts supporting each base. Two kinds of significant deviation occurred. The ones with the highest p-values were due to change of the base presenting the highest counts between at least two isolates. These were all confirmed to be mutations through targeted sequencing. The other significant differences in distribution were due to change in the ratio of frequency between the dominant and the second most encountered base at the site. It only occurred in high coverage regions and the dominant base remained the same. None of the positions having such a profile we tested presented a different base across lines. As we do not have the ancestral genome, we used another approach in which instead of using the CFT073 genome, we used a concatenation of contigs resulting from a *de novo* assembly performed with Velvet software [81]. The same mutations events were detected. Through the SSAHA2 package we also identified small indels (<3bp long) compared to CFT073 genome, but none of them appeared to be specific to a subset of strains, they were all shared by all strains.

Finally we wanted to detect IS transpositions. For that purpose, we used the contigs assembled by Velvet software and looked for IS tails in the border of the produced contigs. A library of *E. coli* IS was recovered from ISFinder library (http://www-is.biotoul.fr/is.html). All IS tails were blasted against the assembled contigs. When a significant match was found, the 100 base pairs flanking the targeted sequence in the contig were blasted against the genome of CFT073. This provided a position of IS insertion element in the genome. A few IS insertions were identified as potentially different among the lines. To further comfort the specificity of IS elements integration, in all strains, we looked for the reads overhanging the suspected site of integration and see if any reads were mosaic (having one part of the chromosome and one part of the IS element border). After such a control a single site appeared as an IS integration specific to some strains. It was confirmed by targeted PCR analysis.

As deletion or other genomic rearrangement should also result in mosaic reads (matching two parts of the genome), we used the previous approach to scan all possible positions in the genome that were enriched in such mosaic reads in a specific subset of strains. In addition to the previous IS element, we found a 5 bp deletion in *ompA* uniquely in isolate 58 which was confirmed by targeted sequencing.

While with these different approaches we have scanned close to 90% of the genome for punctual mutations, small insertion or deletions, or IS integration, our approach is much more limited in repeated regions the genome (although the use of *de novo* assembled contigs suggest that in low copy regions no change were observed). Moreover our approaches, based on the use technology, fail to detect any IS based chromosomal reorganisation such as inversion and gene amplification.

All mutations detected by whole genome sequencing (Solexa) were verified by targeted sequencing using Sanger technology on PCR products or by PCR and agarose gel electrophoresis (for the IS detection) and searched on the other isolates of patient 3. The PCR primers were as follow: *mprA*, forward CATTATCATCCCAACACTGC, reverse ACGCCATAAACAACGTCTCG; *rbsR*, forward AATTTAACGGCGGGTTTGAC, reverse TTCAGCAGCAATGTTCATGC; *ompA*, forward ATCGTCTGGCAACGTCTGGC, reverse GACAAAATCTCCGCACGTGG; *yagX*, forward ACTGGTTGTTATAGACGCCG, reverse CGTCCCGCTCTATATTCATC; *ybdL*, forward AACTGTCTGGCAGCAGACTG, reverse TCACCAGAATATCGCGCTTC; *tktA*, forward GGCTGTAGATCAGCATGGAG, reverse GCAACATGCGAGCATGATCC; IS between *gadA* and *gadW*, forward GCCAGACTGTTTCTGTGTGC, reverse CACGGGAAACTTTGTGCTCT; deletion in *ompA*, forward GGGTTACCCAATCACTGACG, reverse CAGCGAAGACCGGAGAAAC.

### Statistical analysis

In order to determinate if a link between various phenotypes (including RpoS level and 27 protein levels obtained by 2-DE analysis) exists we used a non-parametric test in the line of the Spearman's rank correlation test. There is no reason to *a priori* suspect that links between phenotypes are necessary linear. Values have been sorted (equal ranks are given for equal values, as usual in case of nonparametric analysis). However, according to the special characteristics of the dataset, the classical Pearson's rank correlation test cannot be applied here. Indeed, because the obtained values for each patient are not comparable, a unique sorting of data cannot be reached. We thus sort values for each patient. For a couple of phenotypes, the sum of absolute differences between the whole resulting ranks is related to the possible link between phenotypes (the strongest is the link, the lowest is the sum). A permutation test gives access to a *p*-value: ranks are sorted (by patient) in order to evaluate the distribution of such ranks in case of independence between phenotypes (10,000 permutations in our implementation). A similar analysis while ranks are reversed for one phenotype allows accounting of negative links between phenotypes. For each couple of phenotypes, we are thus able to evaluate the probability that a link is existing. Links detected as significant (at the 5% level) are drawn in Figs 6 and S1.

A distance tree of the 8 isolates of patient 3 has been constructed from the phenotype and proteomic data. Because measures of variables cannot be compared, we have rather considered ranks (just as it has been done for the statistical analysis). The tree is performed by neighbour-joining from Euclidean distances on ranks.

### Mathematical modelling

The evolutionary model tracking the rate of change of different phenotypes within the *E. coli* population can be schematically represented as in Fig. S4 and mathematically written as:
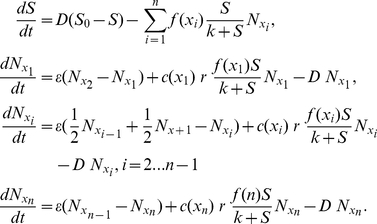
(1)For simplicity we consider a homogeneous environment containing a single limiting resource *S* and assume that bacterial growth on *S* is proportional to the rate of ATP production with *r* denoting the number of cells created per unit of resource and *k* representing the measure of affinity for the resource (for details see [82]). We also make a simplifying assumption that the resource (*S_0_*) diffuses into the system at the same constant rate *D* as the rate at which both resources and bacteria are depleted from the system. As mentioned above these simplifying assumptions regarding the environment and cell metabolism enable us to isolate the effects of SPANC balance trade-off on evolution of diversity of RpoS levels within an *E. coli* population.

## Supporting Information

Figure S1 Statistically significant links between phenotypes. The links are depicted by a line with the *p*-value. The sign of the link (positive in green, phenotypes increase together; or negative in red, a phenotype increases when the other decreases) is also provided. The used data set corresponds to 23 isolates originating from 8 patients.
(0.22 MB TIF)

Figure S2 Level of RpoS studied by immunoblot in the 7 *E. coli* isolates of patient 13 (A) and the 9 *E. coli* isolates of patient 17 (B). RpoS amount is expressed as the ratio of the RpoS band to a constant cross-reactive band. The negative control is *E. coli* MG1655 Δ*rpoS* strain. Experiments were repeated 2 times, given values are the mean of the two experiments.
(0.89 MB TIF)

Figure S3 2-DE gel image of the mix of protein extracts from the 8 representative *E. coli* isolates of patient 3, harvested at the beginning of stationary phase in LB. Proteins were separated by analytical 2-DE and detected by colloidal Coomassie blue staining. The 27 proteins differentially expressed between the 8 isolates are indicated in red. ND corresponds to the putative outer membrane protein. The ranges of the pH in the isoelectrofocalisation and of the molecular weight in the SDS-PAGE are indicated.
(1.80 MB TIF)

Figure S4A schematics of a mathematical model incorporating the SPANC balance trade-off. The model considers an *E. coli* population with *n* competing strains each with a different value of the RpoS expression *x* so that 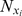 is the density of a strain with phenotype *x_i_* where *i = 1…n* and *0* = *x_1_≤x_2_≤…≤x_n_ = 1*. Evolutionary changes in *x* are constrained by the SPANC balance trade-off in the following way: and increase in *x_i_* leads to a decrease in the maximal resource uptake rate *f(x_i_)* and to an increase in stress protection *c(x_i_)*. Mutations altering *x* occur at a rate *ε*.(0.42 MB TIF)

Table S1 Virulence gene patterns of the *E. coli* isolates belonging to the same clone but exhibiting an intra patient diversity in the virulence gene pattern.
(0.18 MB DOC)

Table S2 Antibiotic resistance patterns of the *E. coli* isolates.
(0.31 MB DOC)

Table S3 Mutator *E. coli* isolates identified by monitoring the isolate capacity to generate mutations conferring resistance to rifampicin.
(0.03 MB DOC)

Table S4 Biolog GN2 microplate results for the *E. coli* isolates from single patients showing an impaired growth.
(0.46 MB DOC)
