# Correction: Molecular and Evolutionary Bases of Within-Patient Genotypic and Phenotypic Diversity in *Escherichia coli* Extraintestinal Infections

**DOI:** 10.1371/annotation/700dcb3b-3475-49a1-9064-d9d070cda7be

**Published:** 2011-06-02

**Authors:** Maxime Levert, Oana Zamfir, Olivier Clermont, Odile Bouvet, Sylvain Lespinats, Marie Claire Hipeaux, Catherine Branger, Bertrand Picard, Claude Saint-Ruf, Françoise Norel, Thierry Balliau, Michel Zivy, Hervé Le Nagard, Stéphane Cruvellier, Béatrice Chane-Woon-Ming, Susanna Nilsson, Ivana Gudelj, Katherine Phan, Thomas Ferenci, Olivier Tenaillon, Erick Denamur

The name of the 14th author was misspelled. The correct spelling is Stéphane Cruveiller.

