# Supplementary figures and images for: Molecular and Evolutionary Bases of Within-Patient Genotypic and Phenotypic Diversity in Escherichia coli Extraintestinal Infections
Source: PLoS Pathog. 2010 Sep 30;6(9):e1001125. doi: 10.1371/journal.ppat.1001125 (PMC2947995; doi:10.1371/journal.ppat.1001125)

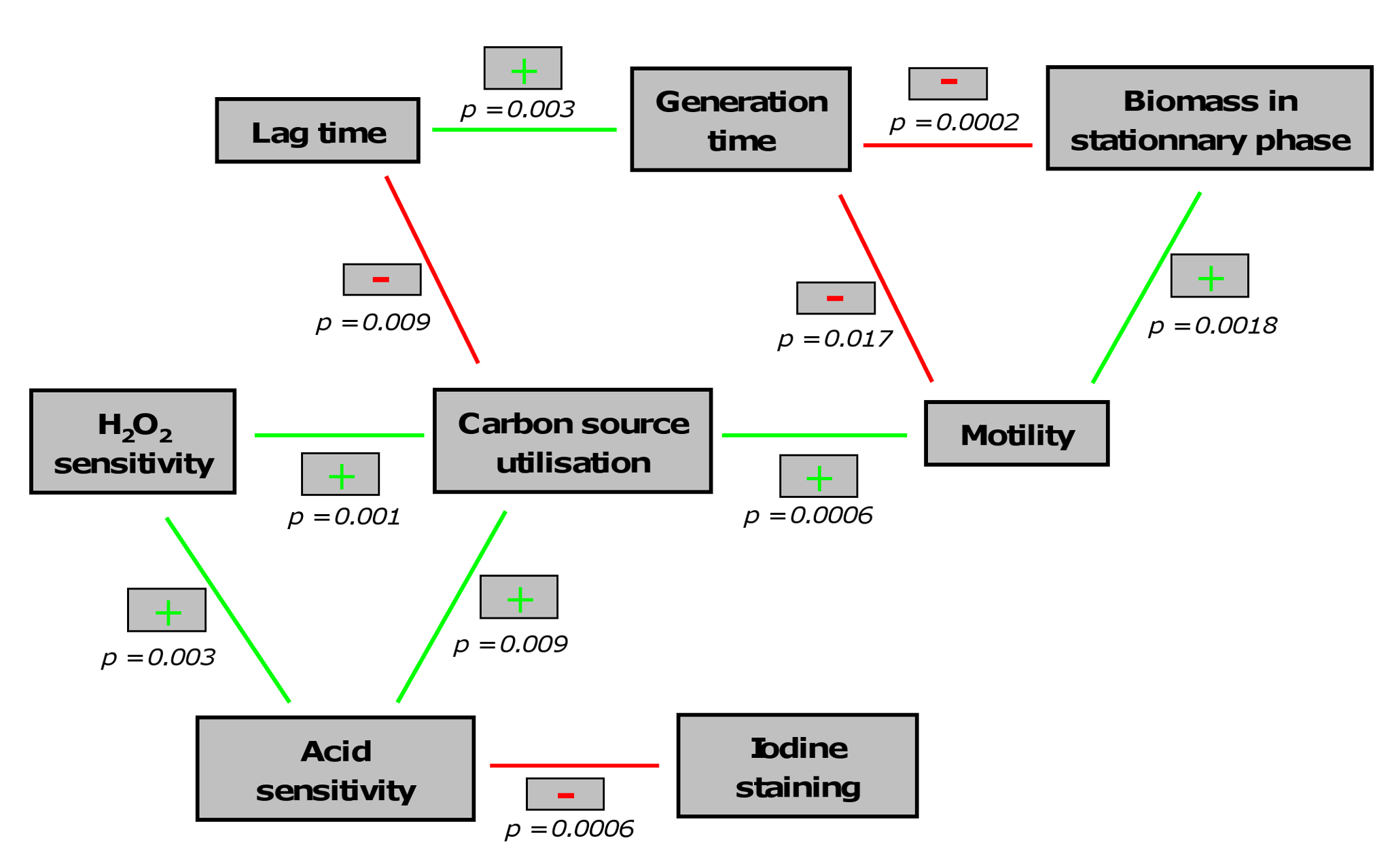

Supplement: Figure S1 — Statistically significant links between phenotypes. The links are depicted by a line with the p-value. The sign of the link (positive in green, phenotypes increase together; or negative in red, a phenotype increases when the other decreases) is also provided. The used data set corresponds to 23 isolates originating from 8 patients. (0.22 MB TIF) [file ppat.1001125.s001.tif]

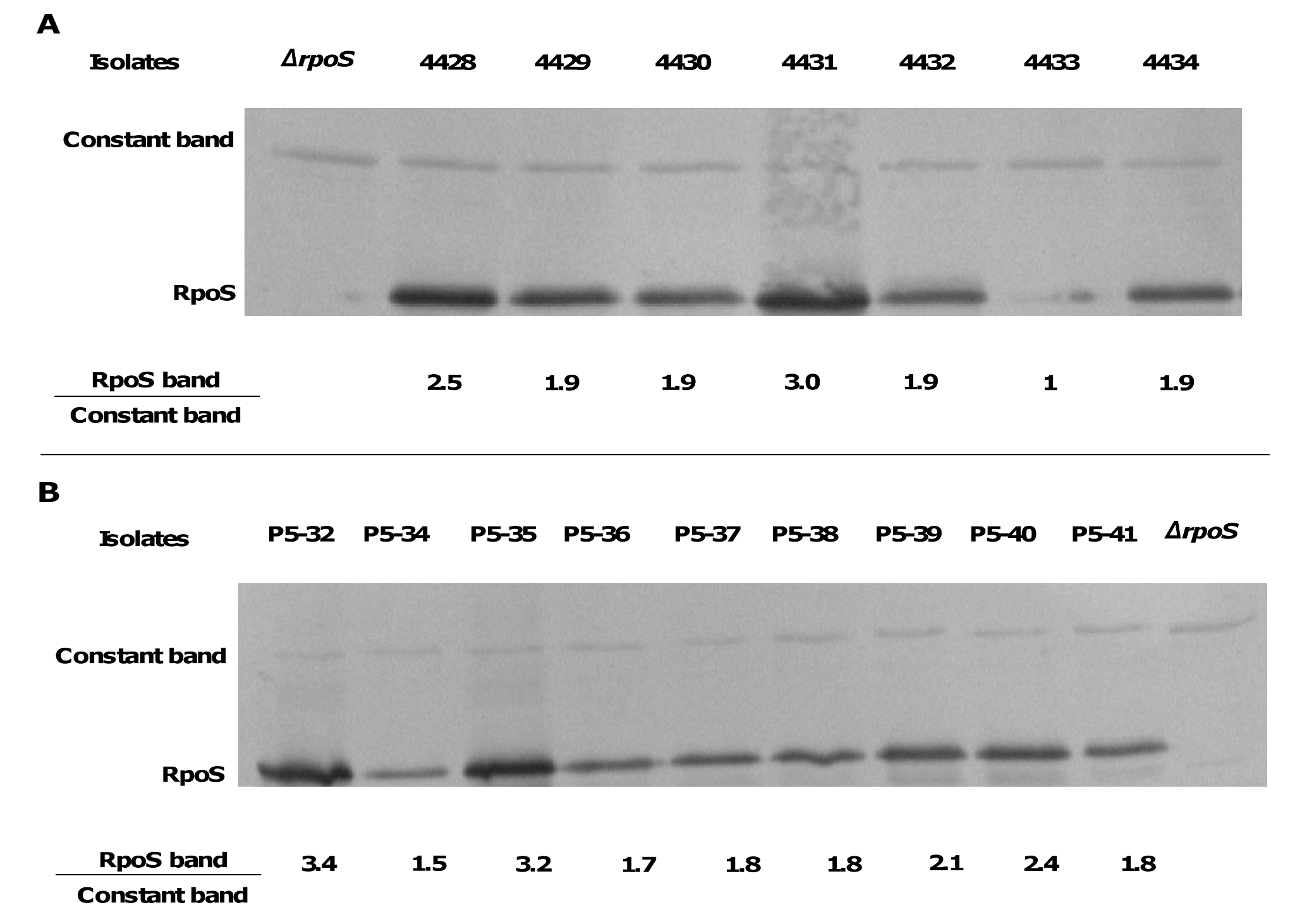

Supplement: Figure S2 — Level of RpoS studied by immunoblot in the 7 E. coli isolates of patient 13 (A) and the 9 E. coli isolates of patient 17 (B). RpoS amount is expressed as the ratio of the RpoS band to a constant cross-reactive band. The negative control is E. coli MG1655 ΔrpoS strain. Experiments were repeated 2 times, given values are the mean of the two experiments. (0.89 MB TIF) [file ppat.1001125.s002.tif]

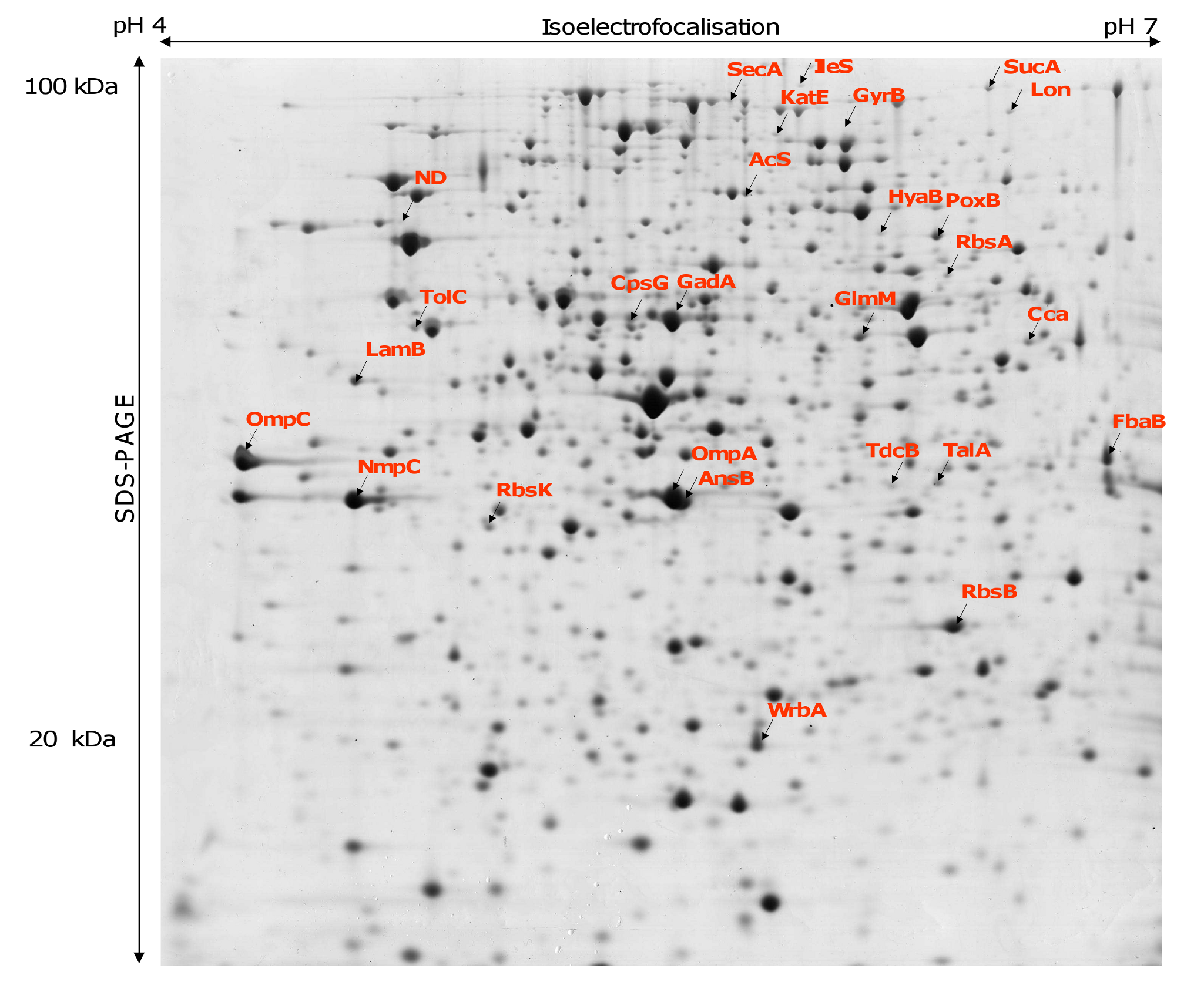

Supplement: Figure S3 — 2-DE gel image of the mix of protein extracts from the 8 representative E. coli isolates of patient 3, harvested at the beginning of stationary phase in LB. Proteins were separated by analytical 2-DE and detected by colloidal Coomassie blue staining. The 27 proteins differentially expressed between the 8 isolates are indicated in red. ND corresponds to the putative outer membrane protein. The ranges of the pH in the isoelectrofocalisation and of the molecular weight in the SDS-PAGE are indicated. (1.80 MB TIF) [file ppat.1001125.s003.tif]

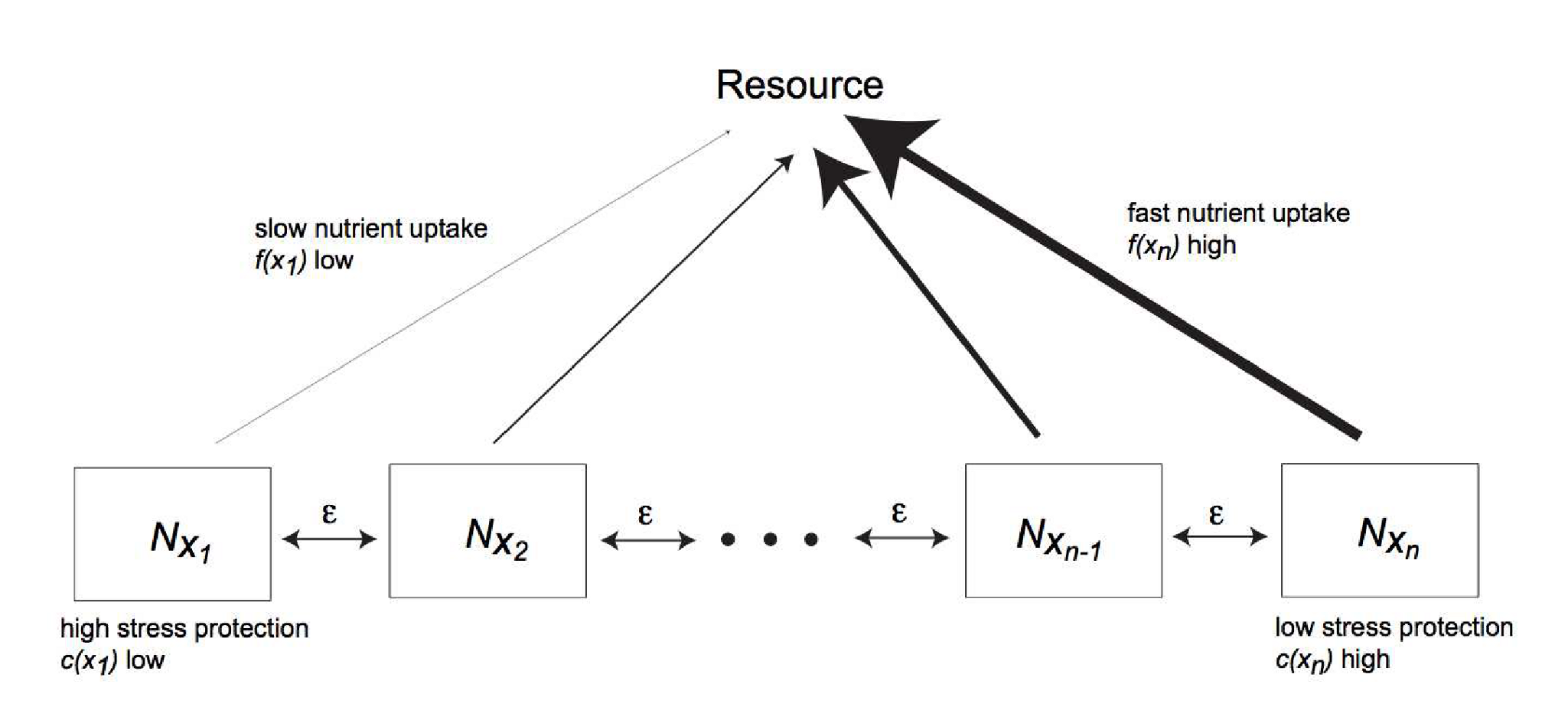

Supplement: Figure S4 — A schematics of a mathematical model incorporating the SPANC balance trade-off. The model considers an E. coli population with n competing strains each with a different value of the RpoS expression x so that is the density of a strain with phenotype xi where i = 1…n and 0 = x1≤x2≤…≤xn = 1. Evolutionary changes in x are constrained by the SPANC balance trade-off in the following way: and increase in xi leads to a decrease in the maximal resource uptake rate f(xi) and to an increase in stress protection c(xi). Mutations altering x occur at a rate ε. (0.42 MB TIF) [file ppat.1001125.s004.tif]
