# Supplementary material for: Molecular and Evolutionary Bases of Within-Patient Genotypic and Phenotypic Diversity in Escherichia coli Extraintestinal Infections
Source: PLoS Pathog. 2010 Sep 30;6(9):e1001125. doi: 10.1371/journal.ppat.1001125 (PMC2947995; doi:10.1371/journal.ppat.1001125)
Supplement: Table S1 — Virulence gene patterns of the E. coli isolates belonging to the same clone but exhibiting an intra patient diversity in the virulence gene pattern. (0.18 MB DOC) [file ppat.1001125.s005.doc]

**Table S1.** Virulence gene patterns of the *E. coli* isolates belonging to the same clone but exhibiting an intra patient diversity in the virulence gene pattern

| Isolate ID^a^ | Phylogenetic group/subgroup^b^ | *neuC* (K1) | *chuA* | *sfa/foc* | *iroN* | *iucC* ^c^ | *iutA*^c^ | *iha* | *papC* | *papG* | *hlyC* | *cnf1* | *hra* | *sat* | *ire* | *usp* | *ompT* | *ibeA* | *malX* | *fyuA* | *irp2* | *traT*^c^ |
| --- | --- | --- | --- | --- | --- | --- | --- | --- | --- | --- | --- | --- | --- | --- | --- | --- | --- | --- | --- | --- | --- | --- |
| **14-4231** | B1 | - | - | - | - | + | + | + | - | - | - | - | - | - | - | - | - | - | - | - | - | + |
| **14-4232** | B1 | - | - | - | - | + | + | + | - | - | - | - | - | - | - | - | - | - | - | - | - | + |
| **14-4240** | B1 | - | - | - | - | + | + | + | - | - | - | - | - | - | - | - | - | - | - | - | - | + |
| **14-4241** | B1 | - | - | - | - | + | + | + | - | - | - | - | - | - | - | - | - | - | - | - | - | + |
| **14-4242** | B1 | - | - | - | - | + | + | + | - | - | - | - | - | - | - | - | - | - | - | - | - | + |
| **14-4243** | B1 | - | - | - | - | - | - | + | - | - | - | - | - | - | - | - | - | - | - | - | - | - |
| **14-4244** | B1 | - | - | - | - | + | + | + | - | - | - | - | - | - | - | - | - | - | - | - | - | + |
| **14-4245** | B1 | - | - | - | - | + | + | + | - | - | - | - | - | - | - | - | - | - | - | - | - | + |
| **17-P5-32** | A_1_ | - | - | - | - | + | + | - | + | II | - | - | - | - | - | - | - | - | - | - | - | + |
| **17-P5-34** | A_1_ | - | - | - | - | - | - | - | + | II | - | - | - | - | - | - | - | - | - | - | - | - |
| **17-P5-35** | A_1_ | - | - | - | - | + | + | - | + | II | - | - | - | - | - | - | - | - | - | - | - | + |
| **17-P5-36** | A_1_ | - | - | - | - | + | + | - | + | II | - | - | - | - | - | - | - | - | - | - | - | - |
| **17-P5-37** | A_1_ | - | - | - | - | + | + | - | + | II | - | - | - | - | - | - | - | - | - | - | - | + |
| **17-P5-38** | A_1_ | - | - | - | - | + | + | - | + | II | - | - | - | - | - | - | - | - | - | - | - | + |
| **17-P5-39** | A_1_ | - | - | - | - | + | + | - | + | II | - | - | - | - | - | - | - | - | - | - | - | + |
| **17-P5-40** | A_1_ | - | - | - | - | + | + | - | + | II | - | - | - | - | - | - | - | - | - | - | - | - |
| **17-P5-41** | A_1_ | - | - | - | - | + | + | - | + | II | - | - | - | - | - | - | - | - | - | - | - | + |
| **19-471** | D_1_ | - | + | - | - | - | - | + | + | II | - | - | - | - | + | - | + | - | + | - | - | - |
| **19-472** | D_1_ | - | + | - | - | - | - | + | + | II | - | - | - | - | + | - | + | - | + | - | - | - |
| **19-473** | D_1_ | - | + | - | - | - | - | + | + | II | - | - | - | - | + | - | + | - | + | - | - | - |
| **19-474** | D_1_ | - | + | - | - | - | - | + | + | II | - | - | - | - | + | - | + | - | + | - | - | - |
| **19-475** | D_1_ | - | + | - | - | - | - | + | + | II | - | - | - | - | + | - | + | - | + | - | - | - |
| **19-476** | D_1_ | - | + | - | - | + | + | + | + | II | - | - | - | - | + | - | + | - | + | - | - | + |
| **19-477** | D_1_ | - | + | - | - | + | + | + | + | II | - | - | - | - | + | - | + | - | + | - | - | + |
| **19-478** | D_1_ | - | + | - | - | + | + | + | + | II | - | - | - | - | + | - | + | - | + | - | - | + |
| **19-479** | D_1_ | - | + | - | - | + | + | + | + | II | - | - | - | - | + | - | + | - | + | - | - | + |
| **19-480** | D_1_ | - | + | - | - | + | + | + | + | II | - | - | - | - | + | - | + | - | + | - | - | + |
| **19-481** | D_1_ | - | + | - | - | - | - | + | + | II | - | - | - | - | + | - | + | - | + | - | - | - |
| **19-482** | D_1_ | - | + | - | - | - | - | + | + | II | - | - | - | - | + | - | + | - | + | - | - | - |
| **19-483** | D_1_ | - | + | - | - | - | - | + | + | II | - | - | - | - | + | - | + | - | + | - | - | - |
| **19-484** | D_1_ | - | + | - | - | - | - | + | + | II | - | - | - | - | + | - | + | - | + | - | - | - |
| **19-485** | D_1_ | - | + | - | - | - | - | + | + | II | - | - | - | - | + | - | + | - | + | - | - | - |
| **19-486** | D_1_ | - | + | - | - | + | + | + | + | II | - | - | - | - | + | - | + | - | + | - | - | + |

**^a^** Isolate ID, first number corresponds to the patient ID.

**^b^** Determined as in [63].

**^c^** All these genes are plasmid located.
