# Supplementary material for: Molecular and Evolutionary Bases of Within-Patient Genotypic and Phenotypic Diversity in Escherichia coli Extraintestinal Infections
Source: PLoS Pathog. 2010 Sep 30;6(9):e1001125. doi: 10.1371/journal.ppat.1001125 (PMC2947995; doi:10.1371/journal.ppat.1001125)
Supplement: Table S2 — Antibiotic resistance patterns of the E. coli isolates. (0.31 MB DOC) [file ppat.1001125.s006.doc]

**Table S2**. Antibiotic resistance patterns of the *E. coli* isolates

| Patient ID | Strain ID | Sample | Antibio-type^a^ | AMX^b^ | TIC^b^ | AMX-clavula-nate^b^ | PIP^b^ | PIP-tazo-bactam^b^ | Te^b^ | Mn^b^ | St^b^ | Ka^b^ | Sul^b^ | TMP^b^ | Ch^b^ | Na^b^ |
| --- | --- | --- | --- | --- | --- | --- | --- | --- | --- | --- | --- | --- | --- | --- | --- | --- |
| **3** | 41 | Blood | D | **R** | **R** | S | **R** | S | **R** | **R** | **R** | **R** | **R** | S | S | S |
|  | 42 | Blood | D | **R** | **R** | S | **R** | S | **R** | **R** | **R** | **R** | **R** | S | S | S |
|  | 43 | Blood | D | **R** | **R** | S | **R** | S | **R** | **R** | **R** | **R** | **R** | S | S | S |
|  | 44 | Blood | D | **R** | **R** | S | **R** | S | **R** | **R** | **R** | **R** | **R** | S | S | S |
|  | 45 | Blood | D | **R** | **R** | S | **R** | S | **R** | **R** | **R** | **R** | **R** | S | S | S |
|  | 46 | Blood | D | **R** | **R** | S | **R** | S | **R** | **R** | **R** | **R** | **R** | S | S | S |
|  | 47 | Blood | D | **R** | **R** | S | **R** | S | **R** | **R** | **R** | **R** | **R** | S | S | S |
|  | 48 | Blood | D | **R** | **R** | S | **R** | S | **R** | **R** | **R** | **R** | **R** | S | S | S |
|  | 49 | Blood | D | **R** | **R** | S | **R** | S | **R** | **R** | **R** | **R** | **R** | S | S | S |
|  | 50 | Blood | D | **R** | **R** | S | **R** | S | **R** | **R** | **R** | **R** | **R** | S | S | S |
|  | 51 | Hepatic abscess | E | S | S | S | S | S | **R** | **R** | S | **R** | S | S | S | S |
|  | 52 | Hepatic abscess | D | **R** | **R** | S | **R** | S | **R** | **R** | **R** | **R** | **R** | S | S | S |
|  | 53 | Hepatic abscess | D | **R** | **R** | S | **R** | S | **R** | **R** | **R** | **R** | **R** | S | S | S |
|  | 54 | Hepatic abscess | D | **R** | **R** | S | **R** | S | **R** | **R** | **R** | **R** | **R** | S | S | S |
|  | 55 | Hepatic abscess | D | **R** | **R** | S | **R** | S | **R** | **R** | **R** | **R** | **R** | S | S | S |
|  | 56 | Hepatic abscess | D | **R** | **R** | S | **R** | S | **R** | **R** | **R** | **R** | **R** | S | S | S |
|  | 57 | Hepatic abscess | D | **R** | **R** | S | **R** | S | **R** | **R** | **R** | **R** | **R** | S | S | S |
|  | 58 | Hepatic abscess | D | **R** | **R** | S | **R** | S | **R** | **R** | **R** | **R** | **R** | S | S | S |
|  | 59 | Hepatic abscess | D | **R** | **R** | S | **R** | S | **R** | **R** | **R** | **R** | **R** | S | S | S |
|  | 60 | Hepatic abscess | D | **R** | **R** | S | **R** | S | **R** | **R** | **R** | **R** | **R** | S | S | S |

| Patient ID | Strain ID | Sample | Antibio-  type | AMX | TIC | AMX-clavula-  nate | PIP | PIP-tazo-bactam | Te | Mn | St | Ka | Sul | TMP | Ch | Na |
| --- | --- | --- | --- | --- | --- | --- | --- | --- | --- | --- | --- | --- | --- | --- | --- | --- |
| 8 | 4234 | Bile | C | **R** | **R** | **I** | **R** | **I** | S | S | S | S | S | S | S | S |
|  | 4235 | Bile | C | **R** | **R** | **I** | **R** | **I** | S | S | S | S | S | S | S | S |
|  | 4237 | Bile | C | **R** | **R** | **I** | **R** | **I** | S | S | S | S | S | S | S | S |
|  | 4238 | Bile | A | S | S | S | S | S | S | S | S | S | S | S | S | S |
|  | 4239 | Bile | A | S | S | S | S | S | S | S | S | S | S | S | S | S |
| **11** | 4248 | CSF^c^ | A | S | S | S | S | S | S | S | S | S | S | S | S | S |
|  | 4249 | CSF | B | **HS** | **HS** | **HS** | **HS** | **HS** | S | S | S | S | S | S | S | S |
|  | 4250 | CSF | A | S | S | S | S | S | S | S | S | S | S | S | S | S |
|  | 4251 | CSF | A | S | S | S | S | S | S | S | S | S | S | S | S | S |
|  | 4252 | CSF | A | S | S | S | S | S | S | S | S | S | S | S | S | S |
|  | 4253 | CSF | B | **HS** | **HS** | **HS** | **HS** | **HS** | S | S | S | S | S | S | S | S |
|  | 4254 | CSF | A | S | S | S | S | S | S | S | S | S | S | S | S | S |

| 12 | 4256 | Urine | H | **R** | **R** | **I** | **R** | S | **R** | S | **R** | **R** | **R** | **R** | **R** | **R** |
| --- | --- | --- | --- | --- | --- | --- | --- | --- | --- | --- | --- | --- | --- | --- | --- | --- |
|  | 4258 | Urine | H | **R** | **R** | **I** | **R** | S | **R** | S | **R** | **R** | **R** | **R** | **R** | **R** |
|  | 4259 | Urine | H | **R** | **R** | **I** | **R** | S | **R** | S | **R** | **R** | **R** | **R** | **R** | **R** |
|  | 4260 | Urine | H | **R** | **R** | **I** | **R** | S | **R** | S | **R** | **R** | **R** | **R** | **R** | **R** |
|  | 4261 | Urine | H | **R** | **R** | **I** | **R** | S | **R** | S | **R** | **R** | **R** | **R** | **R** | **R** |
|  | 4262 | Urine | I | S | S | S | S | S | **R** | S | **R** | S | **R** | S | **R** | **R** |

| Patient ID | Strain ID | Sample | Antibio-  type | AMX | TIC | AMX-clavula-  nate | PIP | PIP-tazo-bactam | Te | Mn | St | Ka | Sul | TMP | Ch | Na |
| --- | --- | --- | --- | --- | --- | --- | --- | --- | --- | --- | --- | --- | --- | --- | --- | --- |
| **13** | 4428 | Urine | B | **HS** | **HS** | **HS** | **HS** | **HS** | S | S | S | S | S | S | S | S |
|  | 4429 | Urine | A | S | S | S | S | S | S | S | S | S | S | S | S | S |
|  | 4430 | Urine | A | S | S | S | S | S | S | S | S | S | S | S | S | S |
|  | 4431 | Urine | A | S | S | S | S | S | S | S | S | S | S | S | S | S |
|  | 4432 | Urine | A | S | S | S | S | S | S | S | S | S | S | S | S | S |
|  | 4433 | Urine | A | S | S | S | S | S | S | S | S | S | S | S | S | S |
|  | 4434 | Urine | A | S | S | S | S | S | S | S | S | S | S | S | S | S |
| **14** |  |  |  |  |  |  |  |  |  |  |  |  |  |  |  |  |
|  | 4231 | Pleural fluid | M | S | S | S | S | S | S | S | **R** | S | **R** | S | S | S |
|  | 4232 | Pleural fluid | M | S | S | S | S | S | S | S | **R** | S | **R** | S | S | S |
|  | 4240 | Pleural fluid | O | S | S | S | S | S | **R** | S | **R** | S | **R** | S | S | S |
|  | 4241 | Pleural fluid | A | S | S | S | S | S | S | S | S | S | S | S | S | S |
|  | 4242 | Pleural fluid | A | S | S | S | S | S | S | S | S | S | S | S | S | S |
|  | 4243 | Pleural fluid | N | S | S | S | S | S | **R** | S | S | S | S | S | S | S |
|  | 4244 | Pleural fluid | N | S | S | S | S | S | **R** | S | S | S | S | S | S | S |
|  | 4245 | Pleural fluid | M | S | S | S | S | S | S | S | **R** | S | **R** | S | S | S |

| Patient ID | Strain ID | Sample | Antibio-  type | AMX | TIC | AMX-clavula-  nate | PIP | PIP-tazo-bactam | Te | Mn | St | Ka | Sul | TMP | Ch | Na |
| --- | --- | --- | --- | --- | --- | --- | --- | --- | --- | --- | --- | --- | --- | --- | --- | --- |
| **16** | P5-45 | Appendiceal abscess | A | S | S | S | S | S | S | S | S | S | S | S | S | S |
|  | P5-46 | Appendiceal abscess | K | **R** | **R** | **I** | **R** | **I** | **R** | **R** | S | S | S | S | **R** | S |
|  | P5-48 | Appendiceal abscess | K | **R** | **R** | **I** | **R** | **I** | **R** | **R** | S | S | S | S | **R** | S |
|  | P5-49 | Appendiceal abscess | L | **R** | **R** | **I** | **R** | **I** | **R** | **R** | **R** | S | **R** | S | **R** | S |
|  | P6-1 | Appendiceal abscess | K | **R** | **R** | **I** | **R** | **I** | **R** | **R** | S | S | S | S | **R** | S |
|  | P6-2 | Appendiceal abscess | K | **R** | **R** | **I** | **R** | **I** | **R** | **R** | S | S | S | S | **R** | S |
|  | P6-3 | Appendiceal abscess | K | **R** | **R** | **I** | **R** | **I** | **R** | **R** | S | S | S | S | **R** | S |

| **17** | P5-32 | Peritoneal fluid | A | S | S | S | S | S | S | S | S | S | S | S | S | S |
| --- | --- | --- | --- | --- | --- | --- | --- | --- | --- | --- | --- | --- | --- | --- | --- | --- |
|  | P5-34 | Peritoneal fluid | B | **HS** | **HS** | **HS** | **HS** | **HS** | S | S | S | S | S | S | S | S |
|  | P5-35 | Peritoneal fluid | A | S | S | S | S | S | S | S | S | S | S | S | S | S |
|  | P5-36 | Peritoneal fluid | A | S | S | S | S | S | S | S | S | S | S | S | S | S |
|  | P5-37 | Peritoneal fluid | B | **HS** | **HS** | **HS** | **HS** | **HS** | S | S | S | S | S | S | S | S |
|  | P5-38 | Peritoneal fluid | B | **HS** | **HS** | **HS** | **HS** | **HS** | S | S | S | S | S | S | S | S |
|  | P5-39 | Peritoneal fluid | A | S | S | S | S | S | S | S | S | S | S | S | S | S |
|  | P5-40 | Peritoneal fluid | A | S | S | S | S | S | S | S | S | S | S | S | S | S |
|  | P5-41 | Peritoneal fluid | B | **HS** | **HS** | **HS** | **HS** | **HS** | S | S | S | S | S | S | S | S |

| Patient ID | Strain ID | Sample | Antibio-  type | AMX | TIC | AMX-clavula-  nate | PIP | PIP-tazo-bactam | Te | Mn | St | Ka | Sul | TMP | Ch | Na |
| --- | --- | --- | --- | --- | --- | --- | --- | --- | --- | --- | --- | --- | --- | --- | --- | --- |
| **19** | 471 | Urine | A | S | S | S | S | S | S | S | S | S | S | S | S | S |
|  | 472 | Urine | A | S | S | S | S | S | S | S | S | S | S | S | S | S |
|  | 473 | Urine | A | S | S | S | S | S | S | S | S | S | S | S | S | S |
|  | 474 | Urine | A | S | S | S | S | S | S | S | S | S | S | S | S | S |
|  | 475 | Urine | A | S | S | S | S | S | S | S | S | S | S | S | S | S |
|  | 476 | Blood | D | **R** | **R** | **I** | **I** | S | **R** | **R** | **R** | **R** | **R** | S | S | S |
|  | 477 | Blood | D | **R** | **R** | **I** | **I** | S | **R** | **R** | **R** | **R** | **R** | S | S | S |
|  | 478 | Blood | D | **R** | **R** | **I** | **I** | S | **R** | **R** | **R** | **R** | **R** | S | S | S |
|  | 479 | Blood | D | **R** | **R** | **I** | **I** | S | **R** | **R** | **R** | **R** | **R** | S | S | S |
|  | 480 | Blood | D | **R** | **R** | **I** | **I** | S | **R** | **R** | **R** | **R** | **R** | S | S | S |
|  | 481 | Blood | D | S | S | S | S | S | S | S | S | S | S | S | S | S |
|  | 482 | Blood | A | S | S | S | S | S | S | S | S | S | S | S | S | S |
|  | 483 | Blood | A | S | S | S | S | S | S | S | S | S | S | S | S | S |
|  | 484 | Blood | A | S | S | S | S | S | S | S | S | S | S | S | S | S |
|  | 485 | Blood | A | S | S | S | S | S | S | S | S | S | S | S | S | S |
|  | 486 | Blood | D | **R** | **R** | **I** | **I** | S | **R** | **R** | **R** | **R** | **R** | S | S | S |

^a^ Definition of the antibiotype is given in the legend of the Fig. 3.

^b^ Abbreviations of the antibiotics are as defined in Material and Methods.

^c^ CSF: cerebrospinal fluid.
