# Supplementary material for: Molecular and Evolutionary Bases of Within-Patient Genotypic and Phenotypic Diversity in Escherichia coli Extraintestinal Infections
Source: PLoS Pathog. 2010 Sep 30;6(9):e1001125. doi: 10.1371/journal.ppat.1001125 (PMC2947995; doi:10.1371/journal.ppat.1001125)
Supplement: Table S3 — Mutator E. coli isolates identified by monitoring the isolate capacity to generate mutations conferring resistance to rifampicin. (0.03 MB DOC) [file ppat.1001125.s007.doc]

**Table S3**. Mutator *E. coli* isolates identified by monitoring the isolate capacity to generate mutations conferring resistance to rifampicin

| Isolate ID^a^ | Mutation frequency (median) | Strength of mutator^b^ |
| --- | --- | --- |
| 3-51 | 4.11 X 10^-8^ | 10X |
| 12-4259 | 5.00 X 10^-8^ | 10X |
| 17-P5-36 | 3.37 X 10^-7^ | 50X |

**^a^** First number corresponds to the ID patient.

**^b^** Defined as in [33].
