# Supplementary material for: Molecular and Evolutionary Bases of Within-Patient Genotypic and Phenotypic Diversity in Escherichia coli Extraintestinal Infections
Source: PLoS Pathog. 2010 Sep 30;6(9):e1001125. doi: 10.1371/journal.ppat.1001125 (PMC2947995; doi:10.1371/journal.ppat.1001125)
Supplement: Table S4 — Biolog GN2 microplate results for the E. coli isolates from single patients showing an impaired growth. (0.46 MB DOC) [file ppat.1001125.s008.doc]

**Table S4.** Biolog GN2 microplate results for the *E. coli* isolates from single patients showing an impaired growth

| Carbon source^a^ | 3-42 ^b^ | 3-45 | 3-47 | 3-50 | 3-51 | 3-52 | 3-54 | 3-58 | 5-4228 | 5-4225 | 13-4432 | 13-4433 | 15-P5-30 | 15-P5-24 | 17-P5-40 | 17-P5-38 | 17-P5-41 | 19-486 | 19-474 |
| --- | --- | --- | --- | --- | --- | --- | --- | --- | --- | --- | --- | --- | --- | --- | --- | --- | --- | --- | --- |
| α-Cyclodextrin |  |  |  |  |  |  |  |  |  |  |  |  |  |  |  |  |  |  |  |
| **Dextrin** |  |  |  |  |  |  |  |  |  |  |  |  |  |  |  |  |  |  |  |
| Glycogen |  |  |  |  |  |  |  |  |  |  |  |  |  |  |  |  |  |  |  |
| **Tween 40** |  |  |  |  |  |  |  |  |  |  |  |  |  |  |  |  |  |  |  |
| Tween 80 |  |  |  |  |  |  |  |  |  |  |  |  |  |  |  |  |  |  |  |
| N-Acetyl-D-Galactosamine |  |  |  |  |  |  |  |  |  |  |  |  |  |  |  |  |  |  |  |
| N-Acetyl-D-Glucosamine |  |  |  |  |  |  |  |  |  |  |  |  |  |  |  |  |  |  |  |
| Adonitol |  |  |  |  |  |  |  |  |  |  |  |  |  |  |  |  |  |  |  |
| **L-Arabinose** |  |  |  |  |  |  |  |  |  |  |  |  |  |  |  |  |  |  |  |
| **D-Arabitol** |  |  |  |  |  |  |  |  |  |  |  |  |  |  |  |  |  |  |  |
| D-Cellobiose |  |  |  |  |  |  |  |  |  |  |  |  |  |  |  |  |  |  |  |
| I-Erythritol |  |  |  |  |  |  |  |  |  |  |  |  |  |  |  |  |  |  |  |
| **D-Fructose** |  |  |  |  |  |  |  |  |  |  |  |  |  |  |  |  |  |  |  |
| L-Fucose |  |  |  |  |  |  |  |  |  |  |  |  |  |  |  |  |  |  |  |
| **D-Galactose** |  |  |  |  |  |  |  |  |  |  |  |  |  |  |  |  |  |  |  |
| Gentibiose |  |  |  |  |  |  |  |  |  |  |  |  |  |  |  |  |  |  |  |
| α-D-Glucose |  |  |  |  |  |  |  |  |  |  |  |  |  |  |  |  |  |  |  |
| m-inositol |  |  |  |  |  |  |  |  |  |  |  |  |  |  |  |  |  |  |  |
| α-D-Lactose |  |  |  |  |  |  |  |  |  |  |  |  |  |  |  |  |  |  |  |
| **Lactulose** |  |  |  |  |  |  |  |  |  |  |  |  |  |  |  |  |  |  |  |
| Maltose |  |  |  |  |  |  |  |  |  |  |  |  |  |  |  |  |  |  |  |
| D-Mannitol |  |  |  |  |  |  |  |  |  |  |  |  |  |  |  |  |  |  |  |
| D-Mannose |  |  |  |  |  |  |  |  |  |  |  |  |  |  |  |  |  |  |  |
| **D-Melibiose*** |  |  |  |  |  |  |  |  |  |  |  |  |  |  |  |  |  |  |  |
| β-Methyl-D-Glucoside* |  |  |  |  |  |  |  |  |  |  |  |  |  |  |  |  |  |  |  |
| D-Psicose |  |  |  |  |  |  |  |  |  |  |  |  |  |  |  |  |  |  |  |
| D-Raffinose |  |  |  |  |  |  |  |  |  |  |  |  |  |  |  |  |  |  |  |
| L-Rhamnose* |  |  |  |  |  |  |  |  |  |  |  |  |  |  |  |  |  |  |  |
| **D-Sorbitol*** |  |  |  |  |  |  |  |  |  |  |  |  |  |  |  |  |  |  |  |
| Sucrose |  |  |  |  |  |  |  |  |  |  |  |  |  |  |  |  |  |  |  |
| D-Trehalose |  |  |  |  |  |  |  |  |  |  |  |  |  |  |  |  |  |  |  |
| **Turanose** |  |  |  |  |  |  |  |  |  |  |  |  |  |  |  |  |  |  |  |
| Xylitol |  |  |  |  |  |  |  |  |  |  |  |  |  |  |  |  |  |  |  |
| **Pyruvic Acid Mono-Methyl Ester** |  |  |  |  |  |  |  |  |  |  |  |  |  |  |  |  |  |  |  |
| **Succinic Acid Mono-Methyl-Ester** |  |  |  |  |  |  |  |  |  |  |  |  |  |  |  |  |  |  |  |
| **Acetic Acid*** |  |  |  |  |  |  |  |  |  |  |  |  |  |  |  |  |  |  |  |

| Carbon source^a^ | 3-42 | 3-45 | 3-47 | 3-50 | 3-51 | 3-52 | 3-54 | 3-58 | 5-4228 | 5-4225 | 13-4432 | 13-4433 | 15-P5-30 | 15-P5-24 | 17-P5-40 | 17-P5-38 | 17-P5-41 | 19-486 | 19-474 |
| --- | --- | --- | --- | --- | --- | --- | --- | --- | --- | --- | --- | --- | --- | --- | --- | --- | --- | --- | --- |
| Cis-Aconitic Acid |  |  |  |  |  |  |  |  |  |  |  |  |  |  |  |  |  |  |  |
| Citric Acid |  |  |  |  |  |  |  |  |  |  |  |  |  |  |  |  |  |  |  |
| Formic Acid |  |  |  |  |  |  |  |  |  |  |  |  |  |  |  |  |  |  |  |
| D-Galactonic Acid Lactone |  |  |  |  |  |  |  |  |  |  |  |  |  |  |  |  |  |  |  |
| D-Galacturonic Acid* |  |  |  |  |  |  |  |  |  |  |  |  |  |  |  |  |  |  |  |
| D-Gluconic Acid |  |  |  |  |  |  |  |  |  |  |  |  |  |  |  |  |  |  |  |
| D-Glucosaminic Acid |  |  |  |  |  |  |  |  |  |  |  |  |  |  |  |  |  |  |  |
| D-Glucuronic Acid |  |  |  |  |  |  |  |  |  |  |  |  |  |  |  |  |  |  |  |
| α-Hydroxybutyric Acid |  |  |  |  |  |  |  |  |  |  |  |  |  |  |  |  |  |  |  |
| Β-Hydroxybutyric Acid |  |  |  |  |  |  |  |  |  |  |  |  |  |  |  |  |  |  |  |
| γ-Hydroxybutyric Acid |  |  |  |  |  |  |  |  |  |  |  |  |  |  |  |  |  |  |  |
| p-Hydroxy Phenylacetic Acid |  |  |  |  |  |  |  |  |  |  |  |  |  |  |  |  |  |  |  |
| Itaconic Acid |  |  |  |  |  |  |  |  |  |  |  |  |  |  |  |  |  |  |  |
| α-Keto Butyric Acid |  |  |  |  |  |  |  |  |  |  |  |  |  |  |  |  |  |  |  |
| **α-Keto Glutaric Acid** |  |  |  |  |  |  |  |  |  |  |  |  |  |  |  |  |  |  |  |
| A-Keto Valeric Acid |  |  |  |  |  |  |  |  |  |  |  |  |  |  |  |  |  |  |  |
| D,L-Lactic Acid |  |  |  |  |  |  |  |  |  |  |  |  |  |  |  |  |  |  |  |
| Malonic Acid |  |  |  |  |  |  |  |  |  |  |  |  |  |  |  |  |  |  |  |
| **Propionic Acid** |  |  |  |  |  |  |  |  |  |  |  |  |  |  |  |  |  |  |  |
| Quinic Acid |  |  |  |  |  |  |  |  |  |  |  |  |  |  |  |  |  |  |  |
| D-Saccharic Acid |  |  |  |  |  |  |  |  |  |  |  |  |  |  |  |  |  |  |  |
| Sebacic Acid |  |  |  |  |  |  |  |  |  |  |  |  |  |  |  |  |  |  |  |
| **Succinic Acid*** |  |  |  |  |  |  |  |  |  |  |  |  |  |  |  |  |  |  |  |
| **Bromosuccinic Acid*** |  |  |  |  |  |  |  |  |  |  |  |  |  |  |  |  |  |  |  |
| Succinamic Acid |  |  |  |  |  |  |  |  |  |  |  |  |  |  |  |  |  |  |  |
| **Glucuronamide** |  |  |  |  |  |  |  |  |  |  |  |  |  |  |  |  |  |  |  |
| L-Alaninamide |  |  |  |  |  |  |  |  |  |  |  |  |  |  |  |  |  |  |  |
| **D-Alanine** |  |  |  |  |  |  |  |  |  |  |  |  |  |  |  |  |  |  |  |
| **L-Alanine*** |  |  |  |  |  |  |  |  |  |  |  |  |  |  |  |  |  |  |  |

| Carbon source^a^ | 3-42 | 3-45 | 3-47 | 3-50 | 3-51 | 3-52 | 3-54 | 3-58 | 5-4228 | 5-4225 | 13-4432 | 13-4433 | 15-P5-30 | 15-P5-24 | 17-P5-40 | 17-P5-38 | 17-P5-41 | 19-486 | 19-474 |
| --- | --- | --- | --- | --- | --- | --- | --- | --- | --- | --- | --- | --- | --- | --- | --- | --- | --- | --- | --- |
| L-Alanyl-Glycine* |  |  |  |  |  |  |  |  |  |  |  |  |  |  |  |  |  |  |  |
| **L-Asparagine*** |  |  |  |  |  |  |  |  |  |  |  |  |  |  |  |  |  |  |  |
| **L-Aspartic Acid*** |  |  |  |  |  |  |  |  |  |  |  |  |  |  |  |  |  |  |  |
| **L-Glutamic Acid** |  |  |  |  |  |  |  |  |  |  |  |  |  |  |  |  |  |  |  |
| **Glycyl-L-Aspartic Acid** |  |  |  |  |  |  |  |  |  |  |  |  |  |  |  |  |  |  |  |
| **Glycyl-L-Glutamic Acid** |  |  |  |  |  |  |  |  |  |  |  |  |  |  |  |  |  |  |  |
| L-Histidine |  |  |  |  |  |  |  |  |  |  |  |  |  |  |  |  |  |  |  |
| Hydroxy-L-Proline |  |  |  |  |  |  |  |  |  |  |  |  |  |  |  |  |  |  |  |
| L-Leucine |  |  |  |  |  |  |  |  |  |  |  |  |  |  |  |  |  |  |  |
| L-Ornitine |  |  |  |  |  |  |  |  |  |  |  |  |  |  |  |  |  |  |  |
| L-Phenylalanine |  |  |  |  |  |  |  |  |  |  |  |  |  |  |  |  |  |  |  |
| **L-Proline** |  |  |  |  |  |  |  |  |  |  |  |  |  |  |  |  |  |  |  |
| L-Pyroglutamic Acid |  |  |  |  |  |  |  |  |  |  |  |  |  |  |  |  |  |  |  |
| **D-Serine** |  |  |  |  |  |  |  |  |  |  |  |  |  |  |  |  |  |  |  |
| **L-Serine** |  |  |  |  |  |  |  |  |  |  |  |  |  |  |  |  |  |  |  |
| L-Threonine |  |  |  |  |  |  |  |  |  |  |  |  |  |  |  |  |  |  |  |
| D,L-Carnitine |  |  |  |  |  |  |  |  |  |  |  |  |  |  |  |  |  |  |  |
| γ-Amino Butyric Acid |  |  |  |  |  |  |  |  |  |  |  |  |  |  |  |  |  |  |  |
| Uranic Acid |  |  |  |  |  |  |  |  |  |  |  |  |  |  |  |  |  |  |  |
| Inosine |  |  |  |  |  |  |  |  |  |  |  |  |  |  |  |  |  |  |  |
| Uridine |  |  |  |  |  |  |  |  |  |  |  |  |  |  |  |  |  |  |  |
| Thymidine |  |  |  |  |  |  |  |  |  |  |  |  |  |  |  |  |  |  |  |
| Phenyethylamine |  |  |  |  |  |  |  |  |  |  |  |  |  |  |  |  |  |  |  |
| Putrescine |  |  |  |  |  |  |  |  |  |  |  |  |  |  |  |  |  |  |  |
| 2-Aminoethanol |  |  |  |  |  |  |  |  |  |  |  |  |  |  |  |  |  |  |  |
| 2,3-Butandiol |  |  |  |  |  |  |  |  |  |  |  |  |  |  |  |  |  |  |  |
| **Glycerol** |  |  |  |  |  |  |  |  |  |  |  |  |  |  |  |  |  |  |  |
| **D,L-α-Glycerol Phosphate*** |  |  |  |  |  |  |  |  |  |  |  |  |  |  |  |  |  |  |  |
| α-D-Glucose-1-Phosphate |  |  |  |  |  |  |  |  |  |  |  |  |  |  |  |  |  |  |  |
| D-Glucose-6-Phosphate |  |  |  |  |  |  |  |  |  |  |  |  |  |  |  |  |  |  |  |


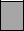
 Substrate use


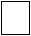
 Absence of substrate use

**^a^** In bold, substrates differentially used between isolates from a single patient.

**^b^** Isolate ID, first number corresponds to the patient ID.

* Substrates used as a source of carbon in *E. coli* MG1655 ∆*rpoS* strains [39].
